# Petasin at the Membrane: Fine-Tuning Lipid Organization and Interfacial Electrostatics in Mast Cell Plasma Membrane Models

**DOI:** 10.3390/molecules31203766

**Published:** 2026-10-20

**Authors:** Vesela Yordanova, Ralitsa Angelova, Rusina Hazarosova, Aleksandrina Nesheva, Dayana Benkova, Tanya Tancheva, Ivaylo Vasilev, Ralitsa Veleva, Dilyana Doneva, Albena Momchilova, Galya Staneva

**Affiliations:** 1 Institute of Biophysics and Biomedical Engineering, Bulgarian Academy of Sciences, Acad. Georgi Bonchev Str., bl. 21, 1113 Sofia, Bulgaria; ralitsa.angelova@biophys.bas.bg (R.A.); rhazarosova@biophys.bas.bg (R.H.); tania.tancheva@biophys.bas.bg (T.T.);; 2 Faculty of Biology, Sofia University ‘St. Kliment Ohridski’, 8 Dragan Tzankov Blvd., 1164 Sofia, Bulgaria; 3 Institute of Plant Physiology and Genetics, Bulgarian Academy of Sciences, Acad. Georgi Bonchev Str., bl. 21, 1113 Sofia, Bulgaria; donevadiliana@gmail.com

**Keywords:** petasin, mast cells, biomimetic membranes, raft domains, lipid order, electrostatic properties

## Abstract

Petasin, a lipophilic sesquiterpene ester isolated from *Petasites hybridus*, exhibits well-documented anti-inflammatory and anti-allergic activities, although its molecular mechanisms of action remain incompletely understood. Here, we investigated its direct effects on membrane organization using biomimetic large unilamellar vesicles (LUVs) modeling resting and activated mast cell plasma membranes. The models comprised phosphatidylcholines (EggPC, PAPC), sphingomyelin (SM), ganglioside GD3, and cholesterol (Chol), with phosphatidylserine (PS) additionally incorporated into the activated membrane model. Simplified liquid-disordered (L_d_) and liquid-ordered (L_o_) lipid systems were used to resolve phase-dependent effects. Membrane properties were assessed by Laurdan generalized polarization, DPH fluorescence anisotropy, DPH-TEMPO fluorescence quenching, di-8-ANEPPS dipole potential measurements, and dynamic light scattering (DLS). Petasin exerted phase- and composition-dependent effects on membrane organization. It increased lipid order in relatively disordered membrane environments, while producing only minor changes in highly ordered L_o_ membranes, revealing a clear dependence of its effects on the initial lipid phase state. In heterogeneous mast cell membrane models exhibiting L_o_/L_d_ phase coexistence, DPH-TEMPO quenching indicated a concentration-dependent increase in the estimated size of ordered lipid nanodomains. In parallel, petasin decreased membrane dipole potential in a concentration-dependent manner, demonstrating modulation of membrane interfacial electrostatic properties. These findings indicate coordinated modulation of lipid packing, lateral membrane organization, and interfacial electrostatics, with the membrane response influenced by lipid composition. DLS measurements showed no evidence of vesicle aggregation or major alterations in vesicle size distribution. Overall, these findings identify petasin as a membrane-active modulator capable of fine-tuning lipid order, lateral membrane organization, and interfacial electrostatics in a phase- and composition-dependent manner. Given the importance of membrane organization and lipid raft dynamics in FcεRI clustering and mast cell activation, these membrane effects may represent an additional biophysical mechanism of the anti-inflammatory and anti-allergic actions of petasin. More broadly, our findings highlight membrane physicochemical organization as a relevant factor in the regulation of mast cell function and provide a basis for further investigation of membrane-mediated mechanisms in allergic and mast cell-associated inflammatory responses.

## 1. Introduction

Allergic diseases are chronic inflammatory disorders characterized by exaggerated immune responses to otherwise harmless environmental allergens. Mast cells are the principal effector cells of allergic reactions. Activation of the high-affinity immunoglobulin E (IgE) receptor (FcεRI) initiates intracellular signaling cascades that culminate in degranulation and the release of inflammatory mediators, including histamine, cytokines, and leukotrienes [1,2]. These signaling events are tightly regulated by the structural organization and physicochemical properties of the plasma membrane.

**Figure 1:**
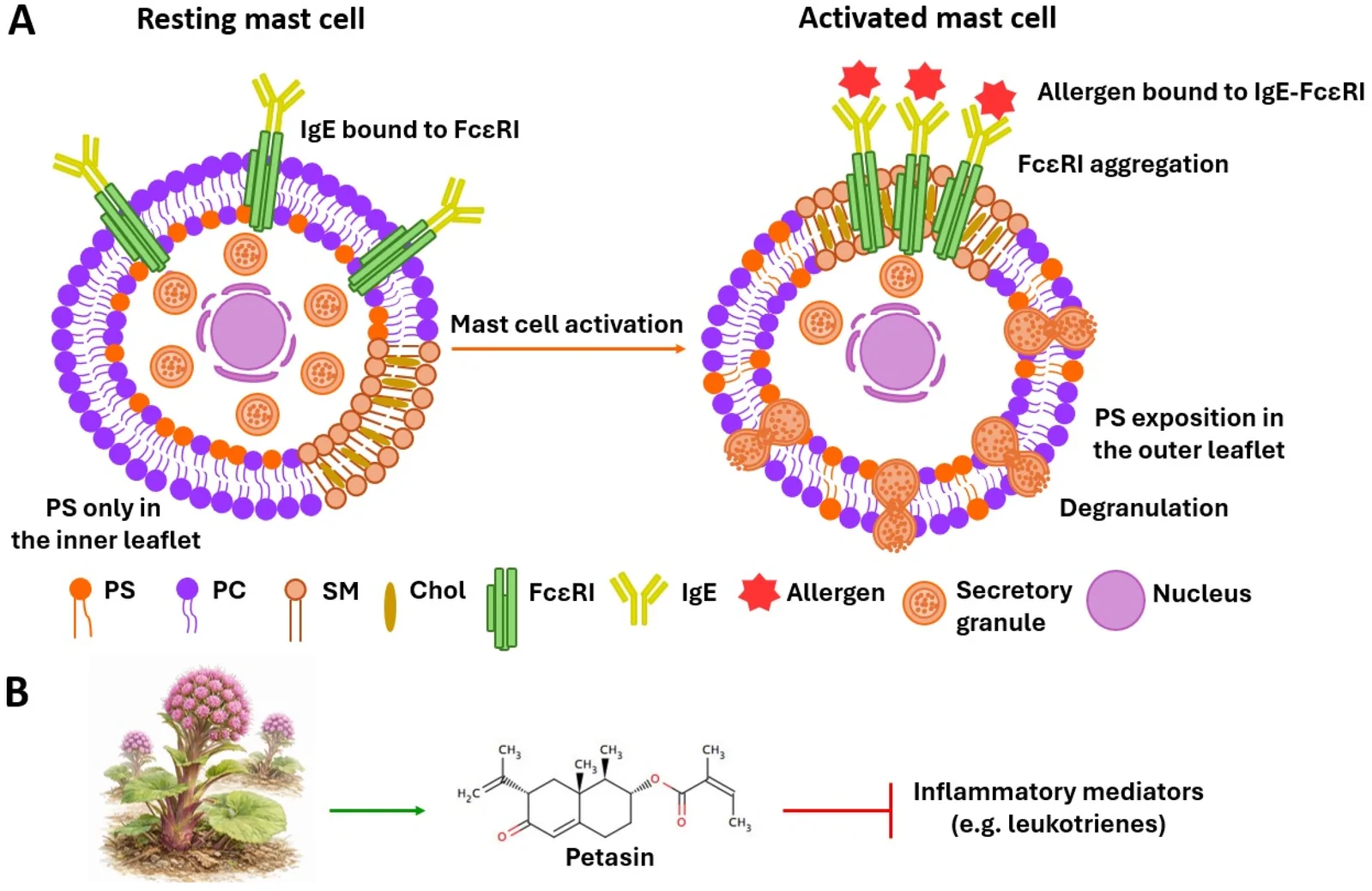
Mast cell activation and petasin-mediated inhibition of leukotriene synthesis. (**A**) Illustration of resting and activated mast cells. Resting mast cells exhibit phosphatidylserine (PS) confined to the inner leaflet, with IgE bound to FcεRI and intact intracellular granules. Upon allergen (Ag)-induced cross-linking of IgE-FcεRI complexes, receptor aggregation triggers activation, accompanied by PS externalization and rapid degranulation, leading to the release of preformed mediators. (**B**) Proposed anti-inflammatory mechanism of petasin, a bioactive constituent of *Petasites hybridus*. Petasin inhibits the formation of cysteinyl leukotrienes (LTC_4_, LTD_4_, and LTE_4_), which are produced through the 5-lipoxygenase (5-LOX) pathway from arachidonic acid released from membrane phospholipids upon cellular activation.

Mast cell plasma membranes contain a complex and dynamic lipid environment in which phospholipid asymmetry, membrane order, and specialized lipid domains coordinate receptor signaling and vesicle trafficking. Phosphatidylserine (PS), an anionic phospholipid predominantly confined to the inner leaflet of resting mast cell membranes, contributes to membrane electrostatics and serves as a platform for the recruitment of signaling proteins [3]. Following allergen-induced FcεRI activation, redistribution of PS together with remodeling of membrane architecture facilitates signaling complex assembly, calcium mobilization, cytoskeletal rearrangement, and membrane fusion during degranulation [4,5]. The transition from resting to activated mast cells, including PS redistribution, FcεRI receptor aggregation, and release of preformed inflammatory mediators, is illustrated in Figure 1A.

A defining feature of mast cell membrane organization is the presence of cholesterol- and sphingolipid-rich raft domains that function as dynamic signaling platforms. Following allergen stimulation, FcεRI and associated signaling proteins, including Lyn kinase, partition into these domains, promoting receptor clustering, tyrosine phosphorylation, and amplification of downstream signaling pathways [6,7]. Consequently, alterations in raft stability, membrane fluidity, and lipid order directly alter mast cell activation and the progression of allergic inflammation.

Petasin, the major bioactive sesquiterpene ester isolated from *Petasites hybridus*, exhibits well-established anti-allergic and anti-inflammatory activities. Its pharmacological activity is primarily attributed to inhibition of the 5-lipoxygenase (5-LOX) pathway, resulting in decreased production of cysteinyl leukotrienes (LTC_4_, LTD_4_, and LTE_4_), which are important mediators of allergic inflammation [8,9]. By limiting leukotriene biosynthesis, petasin reduces inflammatory signaling and contributes to the attenuation of allergic responses. The inhibitory effect of the compound on arachidonic acid metabolism and leukotriene production through suppression of the 5-LOX pathway is presented in Figure 1B.

Although the pharmacological activities of petasin have been extensively investigated, its direct interactions with membrane lipids and their impact on membrane structure remain largely unexplored. The interaction of petasin with biological membranes can be understood in the context of the broader effects of membrane-active natural compounds, which can interact with phospholipid membranes and modify their physicochemical properties. The extent and nature of these effects depend on the molecular characteristics of the compound and the lipid composition of the membrane [10,11]. These findings highlight the importance of investigating the interaction of petasin with lipid membranes. Biomimetic membrane models offer a controlled approach to characterizing its effects on membrane order, lateral lipid organization, and dipole potential, as well as determining whether these changes are accompanied by alterations in vesicle size distribution or colloidal stability.

To our knowledge, no study has systematically characterized these interactions using biomimetic mast cell membrane models in combination with independent biophysical techniques. Since mast cell activation is highly dependent on membrane architecture and lipid-mediated signaling, we hypothesized that petasin modulates the physicochemical properties of mast cell membranes, thereby contributing to its anti-allergic activity through membrane-dependent mechanisms. To test this hypothesis, we examined the effects of petasin on biomimetic large unilamellar vesicles (LUVs) modeling selected features of resting and activated mast cell plasma membranes. Membrane organization was characterized using combined fluorescence-based techniques, including Laurdan generalized polarization, DPH fluorescence anisotropy, DPH-TEMPO fluorescence quenching, and di-8-ANEPPS dipole potential measurements. These analyses were complemented by dynamic light scattering measurements of vesicle size and zeta potential. Examining these parameters can establish a more detailed profile of petasin–membrane interactions and facilitate comparisons with other membrane-active compounds. Although the resulting biophysical changes cannot independently establish the mechanism underlying petasin’s anti-allergic effects, they may provide insights into its interactions with lipid membranes and guide future investigations in cellular models.

## 2. Results

**Figure 2:**
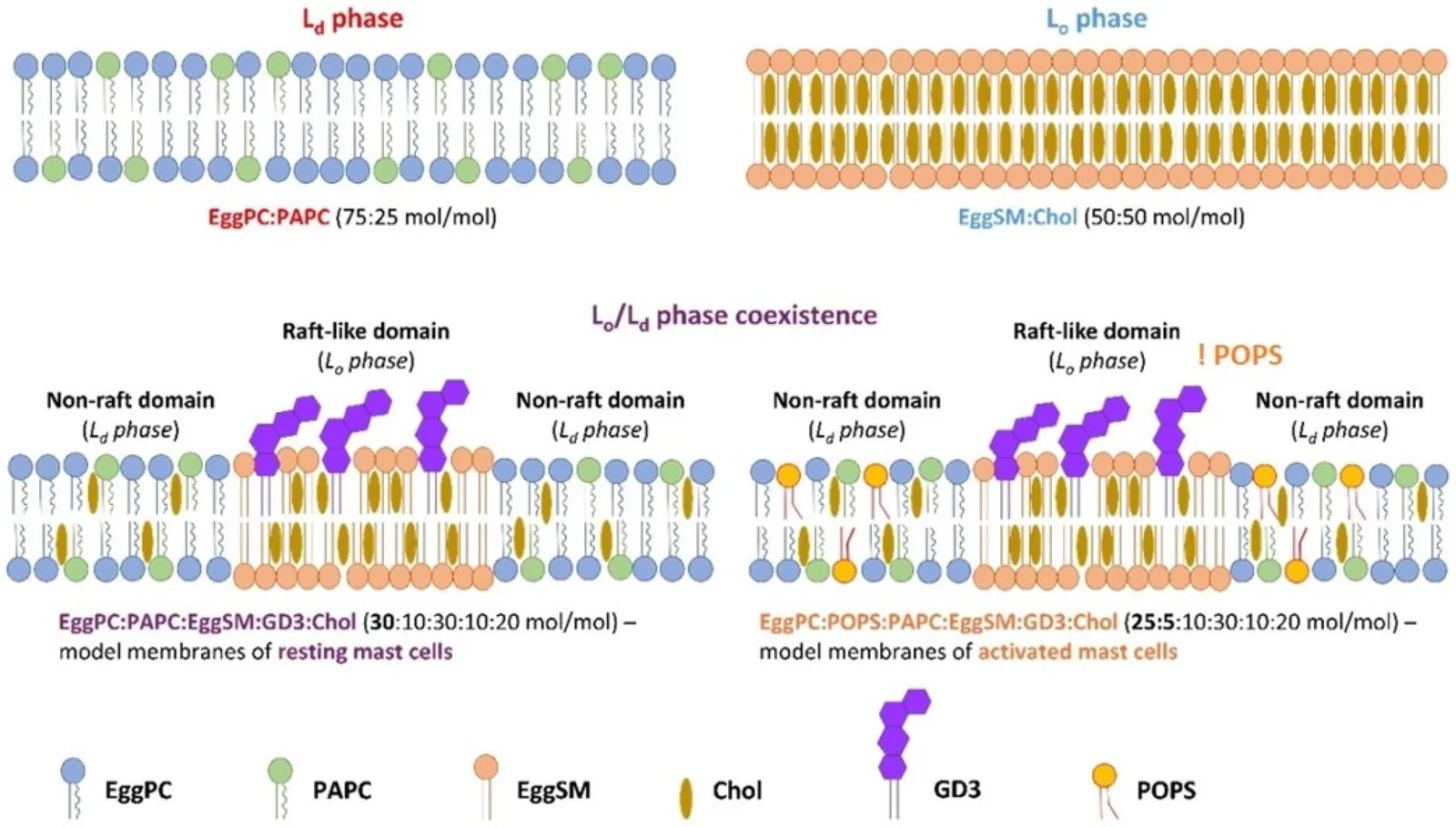
Schematic 2D representation of membrane model systems. Upper: pure liquid-disordered (L_d_, red font) and liquid-ordered (L_o_, blue font) phases. Lower: biomimetic systems with coexisting L_o_/L_d_ (purple font) phases, designed to model selected features of resting (purple font) and activated POPS-containing (orange font) mast cell membranes. Abbreviations and color coding: EggPC, egg phosphatidylcholine (blue, lighter 40%); PAPC, 1-palmitoyl-2-arachidonoyl-*sn*-glycero-3-phosphocholine (green, lighter 40%); EggSM, egg sphingomyelin (orange, lighter 40%); Chol, cholesterol (gold, darker 25%); GD3, disialoganglioside (lavender); POPS, 1-palmitoyl-2-oleoyl-*sn*-glycero-3-phospho-L-serine (orange).

To investigate the membrane-related effects of petasin, a series of multiple biophysical measurements was applied to biomimetic systems (LUVs) representing different membrane phase states and lipid compositions. Three experimental groups were analyzed throughout the study: lipid-only reference samples, DMSO-containing controls, and petasin-treated samples. Petasin was examined at 20 and 50 μM, corresponding to lipid-to-petasin molar ratios of 10:1 and 4:1. These concentrations were selected to ensure measurable interactions with the lipid bilayer while preserving the overall structural properties of the membrane and allowing evaluation of concentration-dependent effects without inducing major changes in vesicle size distribution. The chosen concentration range is commonly employed in membrane biophysics studies of lipophilic bioactive compounds and enables detection of subtle changes in membrane organization and physicochemical properties [12,13,14]. To investigate petasin–membrane interactions in a controlled setting, the principal biomimetic systems were designed to capture selected structural and physicochemical features relevant to resting (EggPC:PAPC:EggSM:GD3:Chol (30:10:30:10:20 mol/mol)) and activated (EggPC:POPS:PAPC:EggSM:GD3:Chol (25:5:10:30:10:20 mol/mol)) mast cell plasma membranes. The lipid components were selected for complementary structural roles: EggPC and PAPC provide a phosphatidylcholine-rich matrix, with polyunsaturated PAPC contributing to a relatively disordered lipid environment; EggSM and Chol favor more ordered, raft-like membrane organization; and GD3 was included as a ganglioside prominently expressed at the mast cell surface. POPS was incorporated into the activated membrane model to introduce an anionic phospholipid component relevant to activation-associated changes in phosphatidylserine distribution and membrane electrostatics [4,5,15]. The use of EggSM and Chol to generate ordered membrane environments is supported by established model-membrane phase behavior [16]. The specific molar ratios used here are defined biomimetic model compositions and are not intended as quantitative reconstructions of the complete mast cell plasma membrane lipidome. Rather, they provide controlled heterogeneous membranes with coexisting ordered and disordered lipid environments for comparison of composition- and phase-dependent effects. In addition, binary EggPC:PAPC (75:25 mol/mol) and EggSM:Chol (50:50 mol/mol) mixtures were used as liquid-disordered (L_d_) and liquid-ordered (L_o_) reference systems, respectively (Figure 2). This experimental design allows phase-dependent responses to be distinguished from those observed in the more complex biomimetic membranes.

### 2.1. Effect of Petasin on Lipid Order at Different Depths of the Membrane Bilayer of Laurdan- and DPH-Labeled LUVs

**Figure 3:**
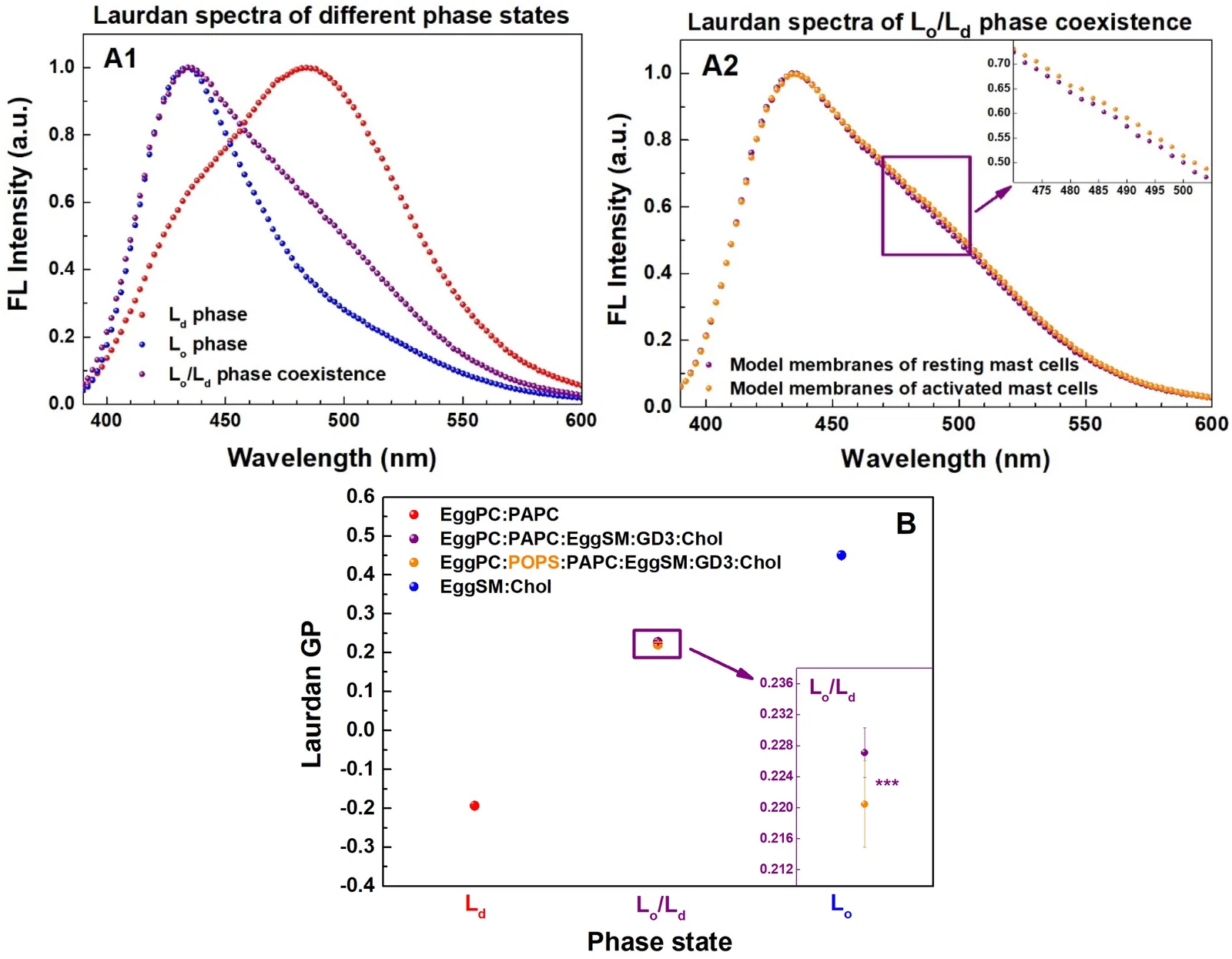
Normalized intensity profiles of fluorescence emission spectra (**A1**,**A2**) and Laurdan GP values (**B**) of binary lipid mixtures, representing the pure liquid-disordered (L_d_, red) and liquid-ordered (L_o_, blue) phases, and complex lipid mixtures, used as biomimetic systems exhibiting L_o_/L_d_ phase coexistence in resting (purple) and activated (orange) mast cells at 37 °C. (**A2**) Inset (indicated by the purple rectangle and arrow): enlarged view of the 470–505 nm spectral region, highlighting the subtle differences between the Laurdan emission spectra of biomimetic systems exhibiting L_o_/L_d_ phase coexistence. (**B**) Inset (indicated by the purple rectangle and arrow): enlarged view of the GP values in the 0.210–0.238 range for biomimetic membranes exhibiting L_o_/L_d_ phase coexistence. Results are presented as mean ± standard deviation (SD) obtained from 3 independent samples with 15 separate measurements per sample (*n* = 45). Laurdan GP values for POPS-containing bilayers (orange sphere) differed significantly from those of POPS-free bilayers (purple sphere) (Pair-Sample *t*-Test, *** *p* < 0.001).

We used Laurdan fluorescence spectroscopy to evaluate the effect of petasin on membrane lipid order at the hydrophobic—hydrophilic interface of biomimetic membrane systems representing different phase states at 37 °C. Laurdan emission spectra clearly distinguished membrane systems representing L_d_, L_o_ and coexisting L_o_/L_d_ membrane phases (Figure 3A1, A2). The EggPC:PAPC (75:25 mol/mol) mixture, representing the L_d_ phase, displayed a red-shifted emission maximum (~490 nm), characteristic of highly fluid and disordered membranes. In contrast, the EggSM:Chol (50:50 mol/mol) mixture, representing the raft-like L_o_ phase, showed a blue-shifted emission maximum (~440 nm), characteristic of tightly packed and highly ordered membranes. The biomimetic membrane models representing resting and activated mast cells exhibited intermediate spectral profiles, consistent with L_o_/L_d_ phase coexistence. Compared with the POPS-free membrane model, incorporation of POPS was accompanied by a modest increase in Laurdan fluorescence intensity in the red spectral region (Figure 3A2, Inset), whereas the blue emission peak remained essentially unchanged.

Generalized polarization (GP) analysis confirmed the differences in membrane lipid order observed in the Laurdan emission spectra (Figure 3B). The EggSM:Chol mixture exhibited the highest GP value (0.450), whereas the EggPC:PAPC mixture displayed the lowest GP value (−0.193). The biomimetic membrane models exhibited intermediate GP values, with the activated mast cell membrane model containing POPS (GP = 0.220) displaying slightly lower membrane order than the corresponding resting membrane model (GP = 0.227) (Figure 3B, Inset).

**Figure 4:**
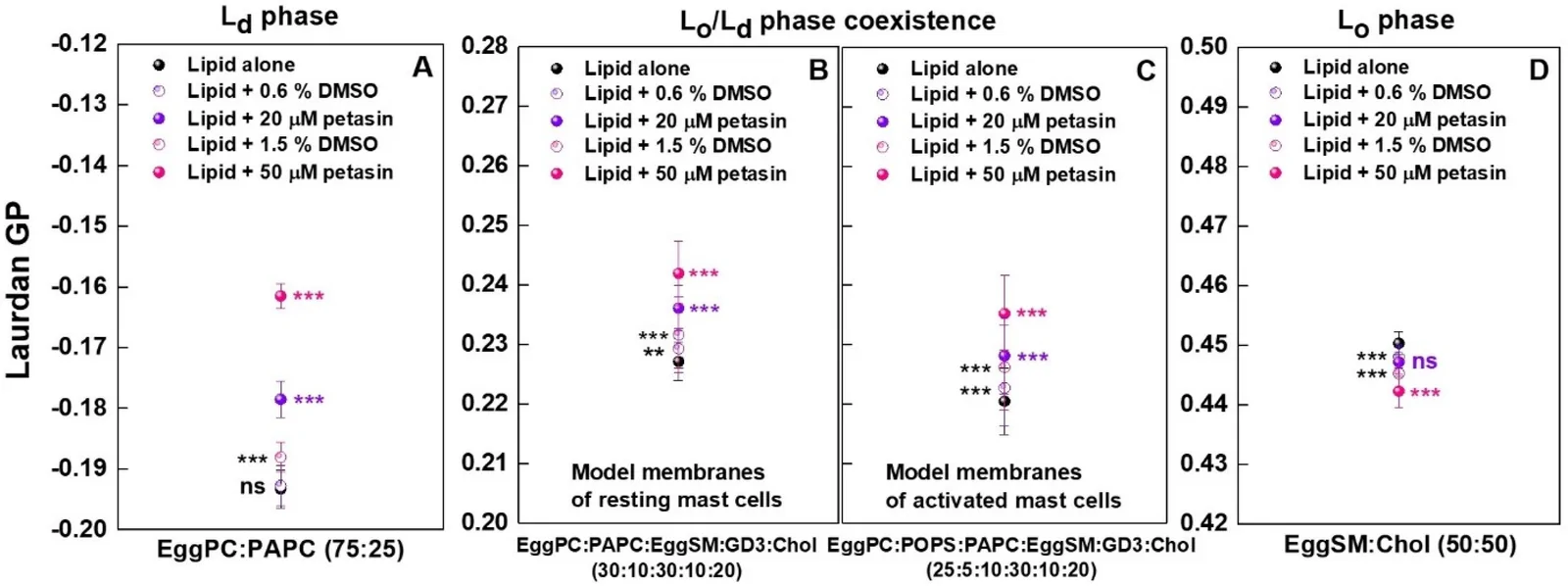
Laurdan GP values of reference samples (lipid alone), DMSO-containing controls, and petasin-treated LUVs at 37 °C. Binary lipid mixtures represent the pure L_d_ (**A**) and L_o_ (**D**) phases, whereas complex lipid mixtures are used as biomimetic systems exhibiting L_o_/L_d_ phase coexistence in resting (**B**) and activated (**C**) mast cells. The effects of petasin at 20 µM (violet, lipid-to-petasin molar ratio 10:1) and 50 µM (pink, lipid-to-petasin molar ratio 4:1) were compared with their corresponding DMSO controls (0.6% and 1.5% DMSO, respectively). Results are presented as mean ± SD obtained from 3 independent samples with 15 measurements per sample (*n* = 45). Pair-Sample *t*-Test was used to assess statistical significance (** *p* < 0.01, *** *p* < 0.001, ns, not significant). Black asterisks indicate significant differences between reference samples (black spheres) and DMSO controls (open spheres), whereas violet and pink asterisks indicate significant differences between petasin-treated samples (solid spheres) and their corresponding DMSO controls (open spheres) at 20 and 50 μM petasin, respectively.

The effect of petasin on membrane lipid order is shown in Figure 4, whereas the corresponding relative (ΔGP, %) and normalized (ΔGP*_norm_*, %) changes are presented in Figure S1A,B. In the EggPC:PAPC membrane, petasin increased GP values by 2.21% and 4.13% at 20 and 50 μM, respectively, demonstrating a concentration-dependent increase in membrane lipid order (Figure 4A and Figure S1B). Similar increases in GP were observed in both biomimetic mast cell membrane models, with petasin-treated samples exhibiting higher GP values than the corresponding DMSO controls (Figure 4B,C). In contrast, petasin-treated EggSM:Chol membranes exhibited a slight decrease in GP relative to the DMSO controls, with normalized changes of −0.14% and −0.46% at 20 and 50 μM petasin, respectively (Figure 4D and Figure S1B). These results demonstrate a small reduction in membrane lipid order in the highly ordered L_o_ phase.

Overall, petasin produced the largest increase in membrane lipid order in the disordered L_d_ membrane, intermediate effects in the biomimetic mast cell membrane models, and only minor changes in the highly ordered L_o_ membrane (Figure S1).

**Figure 5:**
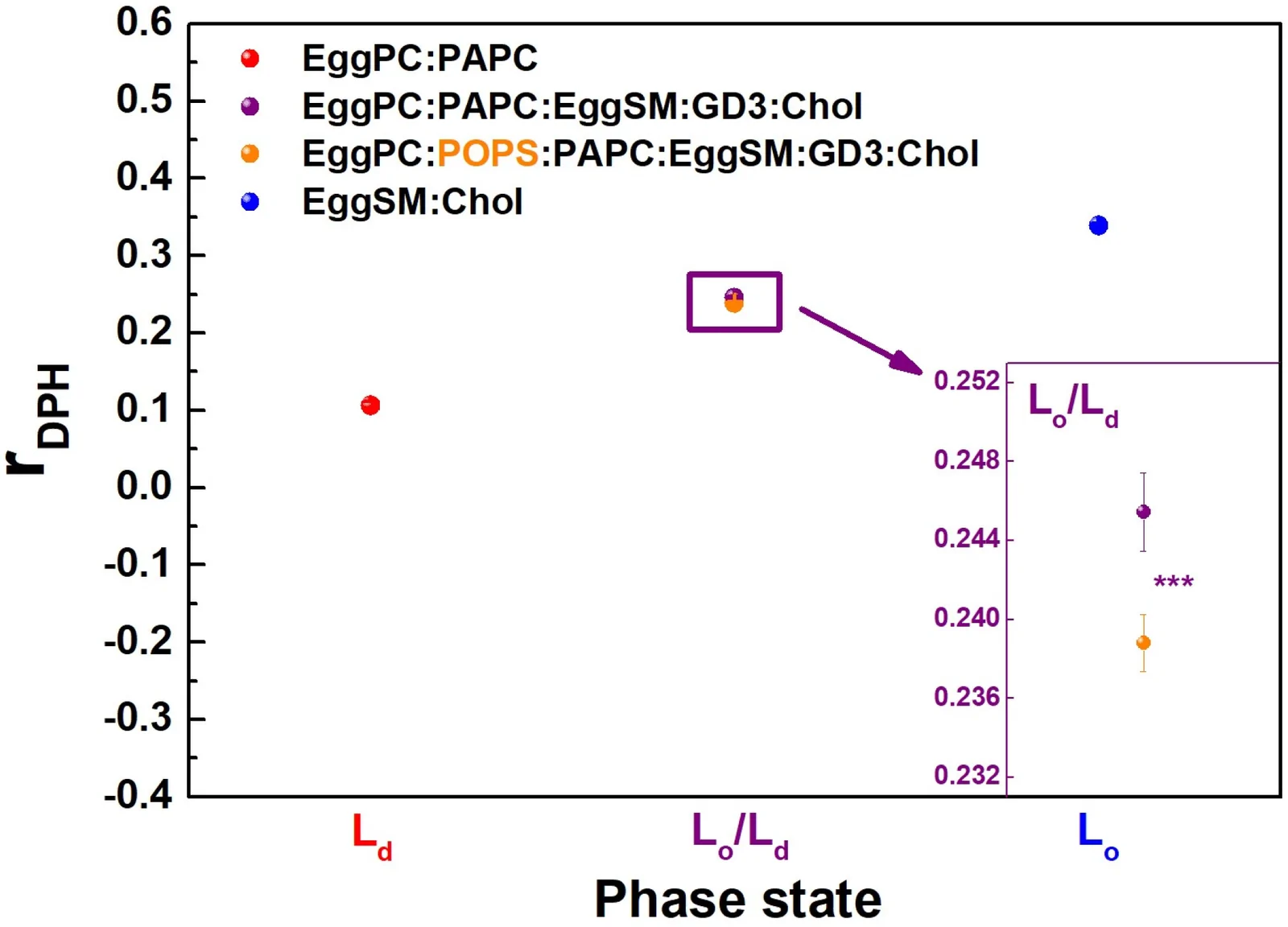
DPH anisotropy (r_DPH_) values of binary lipid mixtures, representing the pure liquid-disordered (L_d_, red) and liquid-ordered (L_o_, blue) phases, and complex lipid mixtures, used as biomimetic systems exhibiting L_o_/L_d_ phase coexistence in resting (purple) and activated (orange) mast cells at 37 °C. Inset (indicated by the purple rectangle and arrow): enlarged view of the r_DPH_ values in the 0.231–0.253 range for biomimetic membranes exhibiting L_o_/L_d_ phase coexistence. Results are presented as mean ± SD obtained from 3 independent samples with 6 measurements per sample (*n* = 18). r_DPH_ values for POPS-containing bilayers (orange sphere) differed significantly from those of POPS-free bilayers (purple sphere) (Pair-Sample *t*-Test, *** *p* < 0.001).

To complement the Laurdan measurements at the membrane interface, DPH fluorescence anisotropy was used to assess lipid order in the hydrophobic core of the membrane bilayer. Because the rotational mobility of DPH is inversely related to membrane fluidity, changes in fluorescence anisotropy (r_DPH_) reflect alterations in acyl-chain packing and membrane order. Figure 5 shows the differences in membrane lipid order among the investigated membrane systems at 37 °C. The EggSM:Chol (50:50 mol/mol) mixture exhibited the highest r_DPH_ value (0.339), consistent with the highly ordered L_o_ phase. In contrast, the EggPC:PAPC (75:25 mol/mol) mixture displayed the lowest r_DPH_ value (0.106), characteristic of the fluid and disordered L_d_ phase. The biomimetic mast cell membrane models exhibited intermediate r_DPH_ values, consistent with L_o_/L_d_ phase coexistence. Compared with the POPS-free membrane model (r_DPH_ = 0.245), incorporation of POPS resulted in a small but statistically significant decrease in r_DPH_ (0.239) (Figure 5, Inset).

**Figure 6:**
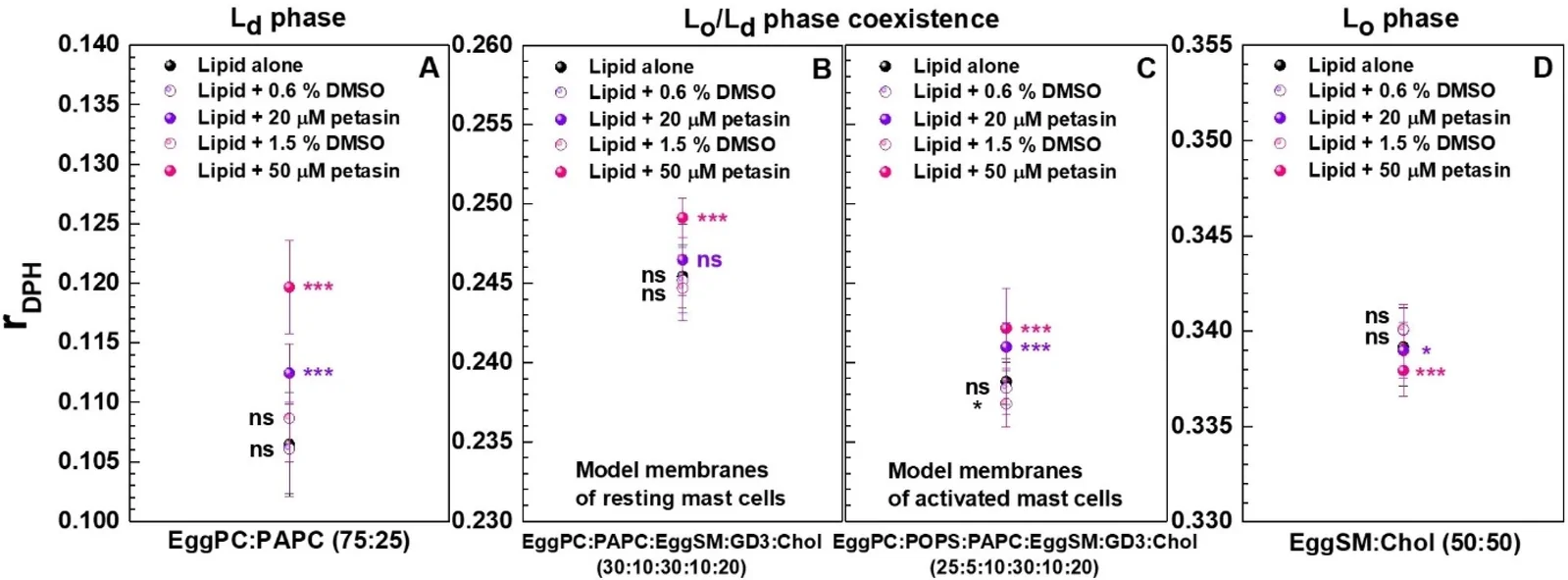
DPH fluorescence anisotropy (r_DPH_) values of reference samples (lipid alone), DMSO-containing controls, and petasin-treated LUVs at 37 °C. Binary lipid mixtures represent the pure L_d_ (**A**) and L_o_ (**D**) phases, whereas complex lipid mixtures are used as biomimetic systems exhibiting L_o_/L_d_ phase coexistence in resting (**B**) and activated (**C**) mast cells. The effects of petasin at 20 µM (violet, lipid-to-petasin molar ratio 10:1) and 50 µM (pink, lipid-to-petasin molar ratio 4:1) were compared with their corresponding DMSO controls (0.6% and 1.5% DMSO, respectively). Results are presented as mean ± SD obtained from 3 independent samples with 6 measurements per sample (*n* = 18). Pair-Sample *t*-Test was used to assess statistical significance (* *p* < 0.05, *** *p* < 0.001, ns, not significant). Black asterisks indicate significant differences between reference samples (black spheres) and DMSO controls (open spheres), whereas violet and pink asterisks indicate significant differences between petasin-treated samples (solid spheres) and their corresponding DMSO controls (open spheres) at 20 and 50 μM petasin, respectively.

The effects of petasin on lipid order within the hydrophobic core were generally in agreement with those observed at the membrane interface by Laurdan fluorescence spectroscopy (Figure 4 and Figure 6). Petasin exhibited concentration- and phase-dependent effects on membrane organization. Similar to the Laurdan measurements, petasin produced the largest effects in membranes with lower initial lipid order, whereas only minor changes were observed in highly ordered membranes. Membranes with lower initial lipid order, including the EggPC:PAPC membrane (Figure 6A) and the biomimetic mast cell membrane models (Figure 6B,C) exhibited increased acyl-chain order following petasin treatment. In contrast, petasin produced a slight decrease in acyl-chain order in the highly ordered EggSM:Chol membrane representing the L_o_ phase (Figure 6D). The magnitude of this decrease increased with petasin concentration.

The corresponding relative (Δr_DPH_, %) and normalized (Δr_DPH_ *_norm_*, %) changes induced by petasin are presented in Figure S2A,B. In the EggPC:PAPC mixture, petasin increased membrane lipid order in a concentration-dependent manner, corresponding to normalized changes of 2.74% and 4.73% at 20 and 50 μM, respectively (Figure S2B). Similar, although less pronounced, increases in membrane lipid order were observed in both biomimetic mast cell membrane models. No statistically significant differences were observed between the effects of petasin at the same concentration (20 or 50 μM) on membrane lipid order in the two heterogeneous model membrane systems representing resting and activated mast cell membranes, either at the hydrophobic-hydrophilic interface, as assessed by Laurdan fluorescence spectroscopy, or within the hydrophobic core of the bilayer, as determined by DPH fluorescence anisotropy (Figures S1B and S2B). In contrast, petasin-treated EggSM:Chol membranes exhibited a slight decrease in membrane lipid order relative to the corresponding DMSO controls, with normalized changes of −0.46% and −0.93% at 20 and 50 μM petasin, respectively (Figure S2B).

Table 1 summarizes the effects of 50 μM petasin on membrane lipid order at two distinct depths of the lipid bilayer, assessed by Laurdan generalized polarization (GP) and DPH fluorescence anisotropy (r_DPH_). Both fluorescence probes exhibited comparable normalized responses across the investigated membrane systems.

### 2.2. Effect of Petasin on Membrane Nanodomain Organization Assessed by DPH-TEMPO Fluorescence Quenching

**Figure 7:**
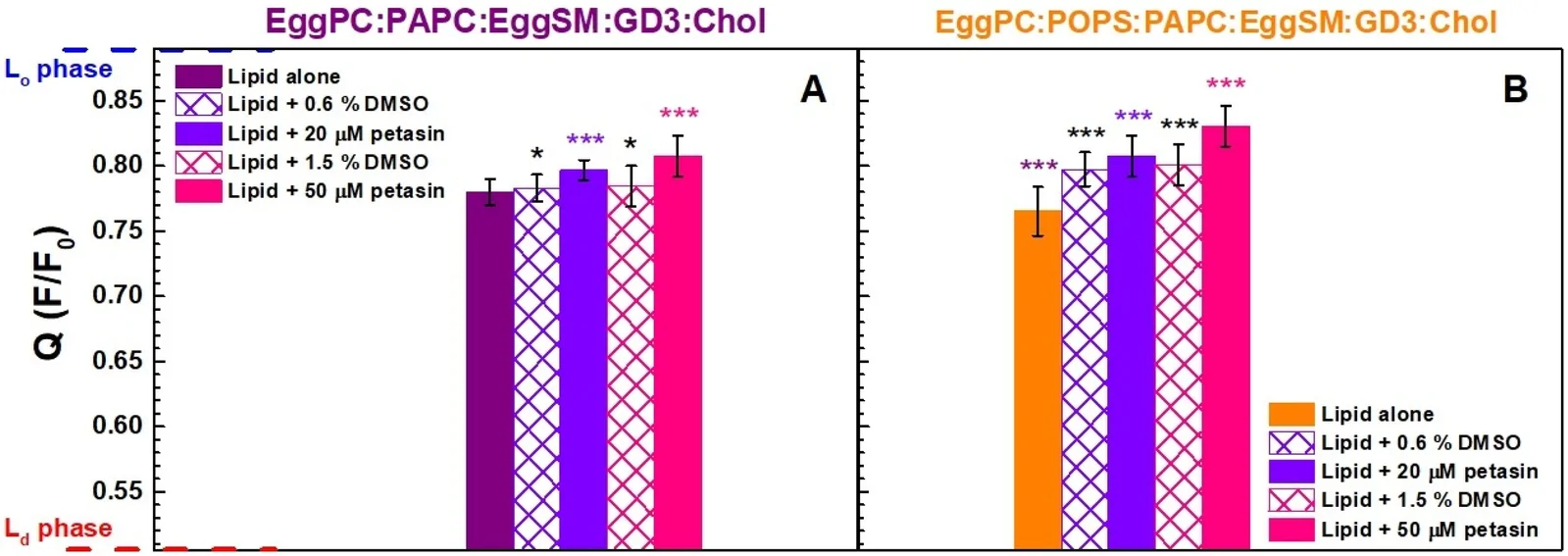
DPH fluorescence quenching by TEMPO expressed as Q (F/F_0_) values in complex lipid mixtures, used as biomimetic systems exhibiting L_o_/L_d_ phase coexistence in (**A**) resting (purple) and (**B**) activated (orange) mast cells. Dashed lines indicate the Q values of binary lipid mixtures, representing the pure liquid-disordered (L_d_, red) and liquid-ordered (L_o_, blue) phases. F and F_0_ denote DPH fluorescence intensity in the presence and absence of TEMPO, respectively. Columns represent reference samples (lipid alone), DMSO controls, and petasin-treated samples at 37 °C. The effects of petasin at 20 μM (violet, lipid-to-petasin molar ratio 10:1) and 50 μM (pink, lipid-to-petasin molar ratio 4:1) were compared with the corresponding DMSO controls (0.6% and 1.5% DMSO, respectively). Results are presented as mean ± SD obtained from 3 independent samples with 15 measurements per sample (*n* = 45). Pair-Sample *t*-test was used to assess statistical significance (* *p* < 0.05, *** *p* < 0.001). Q values for POPS-containing bilayers (orange column) differed significantly from those of POPS-free bilayers (purple column), as indicated by purple asterisks (*** *p* < 0.001). Black asterisks indicate significant differences between reference samples (purple or orange columns) and DMSO controls (sparse-pattern columns), whereas violet and pink asterisks indicate significant differences between petasin-treated samples (solid-pattern columns) and their corresponding DMSO controls (sparse-pattern columns) at 20 and 50 μM petasin, respectively.

DPH fluorescence quenching by TEMPO was employed to assess the relative contribution of ordered and disordered membrane domains in biomimetic membrane models exhibiting L_o_/L_d_ phase coexistence. The Q (F/F_0_) values obtained for the reference samples were intermediate between those measured for the pure L_d_ and L_o_ phases, which accords with the coexistence of ordered and disordered membrane domains in both mast cell model membranes (Figure 7).

The POPS-containing membrane model exhibited significantly lower Q values than the corresponding POPS-free model. In the resting mast cell membrane model, both 0.6% and 1.5% DMSO produced small but statistically significant increases in Q values relative to the reference sample (Figure 7A). Petasin treatment further increased Q values compared with the corresponding DMSO controls at both concentrations. The increase in Q values was concentration-dependent and was greater at 50 μM than at 20 μM petasin. A similar, although more pronounced, response was observed in the activated mast cell membrane model, where DMSO alone produced larger increases in Q values relative to the reference sample (Figure 7B).

The measured Q values were used to estimate the average membrane nanodomain radii (R_dom_) in biomimetic membranes exhibiting L_o_/L_d_ phase coexistence (Table 2). In the absence of petasin, the resting mast cell membrane model exhibited larger estimated nanodomain radii (~34 Å) than the activated POPS-containing membrane model (~32 Å). DMSO alone augmented the estimated nanodomain radii in both membrane systems. This solvent-associated increase was modest in the resting membrane model but considerably greater in the activated membrane model. Relative to the corresponding DMSO controls, petasin produced an additional concentration-dependent increase in the estimated nanodomain radii in both membrane models. In the resting membrane model, petasin increased R_dom_ by approximately 1.7 Å at 20 μM and 2.9 Å at 50 μM. In the activated membrane model, the corresponding increases relative to DMSO were approximately 1.2 Å and 3.7 Å at 20 and 50 μM, respectively. Thus, particularly in the POPS-containing model, the petasin-specific effect should be interpreted against a measurable solvent-dependent background.

### 2.3. Effect of Petasin on Membrane Dipole Potential in di-8-ANEPPS-Labeled LUVs

**Figure 8:**
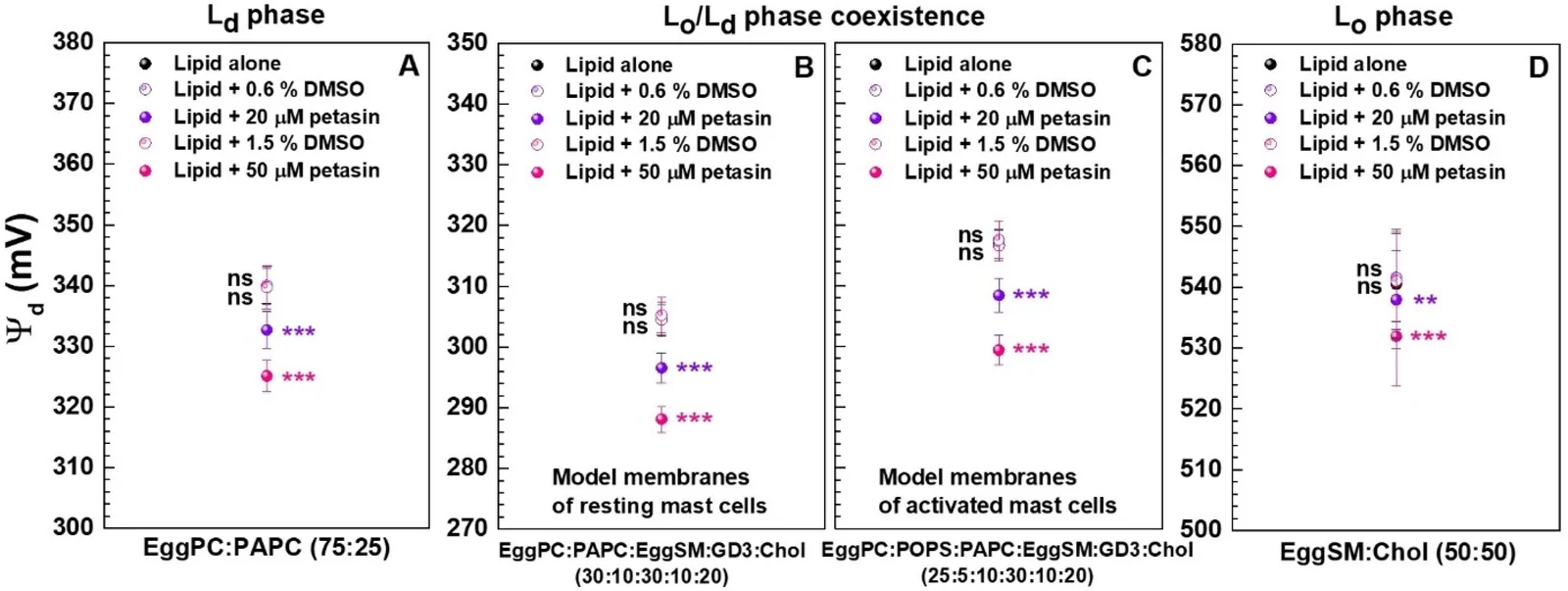
Membrane dipole potential (Ψ_d_) values of reference samples (lipid alone), DMSO controls, and petasin-treated samples at 37 °C. Binary lipid mixtures represent the pure L_d_ (**A**) and L_o_ (**D**) phases, whereas complex lipid mixtures are used as biomimetic systems exhibiting L_o_/L_d_ phase coexistence in resting (**B**) and activated (**C**) mast cells. The effects of petasin at 20 μM (violet, lipid-to-petasin molar ratio 10:1) and 50 μM (pink, lipid-to-petasin molar ratio 4:1) were compared with the corresponding DMSO controls (0.6% and 1.5% DMSO, respectively). Results are presented as mean ± SD obtained from 3 independent samples with 15 measurements per sample (*n* = 45). Pair-Sample *t*-test was used to assess statistical significance (** *p* < 0.01, *** *p* < 0.001; ns, not significant). No statistically significant differences were observed between the reference samples (black spheres) and the corresponding DMSO controls (open spheres), as indicated by “ns”, whereas violet and pink asterisks indicate significant differences between petasin-treated samples (solid spheres) and their corresponding DMSO controls (open spheres) at 20 and 50 μM petasin, respectively.

The effect of petasin on membrane dipole potential (Ψ_d_) is shown in Figure 8, while the corresponding relative (ΔΨ_d_, %) and normalized (ΔΨ_d_ *_norm_*, %) changes are presented in Figure S3 and summarized in Table 3. Reference samples exhibited distinct Ψ_d_ values depending on membrane phase state, with the highest value observed for the highly ordered EggSM:Chol membrane (L_o_ phase) and the lowest for the EggPC:PAPC membrane (L_d_ phase). The biomimetic mast cell membrane models exhibited intermediate Ψ_d_ values characteristic of membranes displaying L_o_/L_d_ phase coexistence.

DMSO controls produced no significant changes in membrane dipole potential compared to the corresponding lipid-alone reference samples (Figure 8; Table 3). In contrast, petasin produced a concentration-dependent decrease in Ψ_d_ across all investigated membrane systems. In the binary EggPC:PAPC LUVs, petasin reduced Ψ_d_ by 3.64% and 7.27% at 20 and 50 μM, respectively (ΔΨ_d_ *_norm_*) (Figure S3B; Table 3). Comparable, although more pronounced, reductions were observed in both mast cell membrane models exhibiting L_o_/L_d_ phase coexistence. In the resting mast cell membrane model, ΔΨ_d_ *_norm_* values decreased by 3.96% and 8.55% at 20 and 50 μM petasin, respectively. Similarly, in the activated POPS-containing membrane model, petasin decreased Ψ_d_ by 4.07% and 9.05% at the corresponding concentrations. At 50 μM petasin, the normalized decrease in Ψ_d_ differed significantly between the two mast cell membrane models (Figure S3B). The highly ordered EggSM:Chol LUVs showed the smallest changes in dipole potential following petasin incorporation, with normalized decreases of 1.81% and 4.56% at 20 and 50 μM, respectively.

Overall, petasin produced a concentration-dependent decrease in membrane dipole potential across all investigated membrane systems. The largest normalized decreases were observed in the biomimetic mast cell membrane models exhibiting L_o_/L_d_ phase coexistence, whereas the smallest changes were detected in the EggSM:Chol membrane.

### 2.4. Effect of Petasin on Vesicle Size and Surface Electrostatic Properties of LUVs

**Figure 9:**
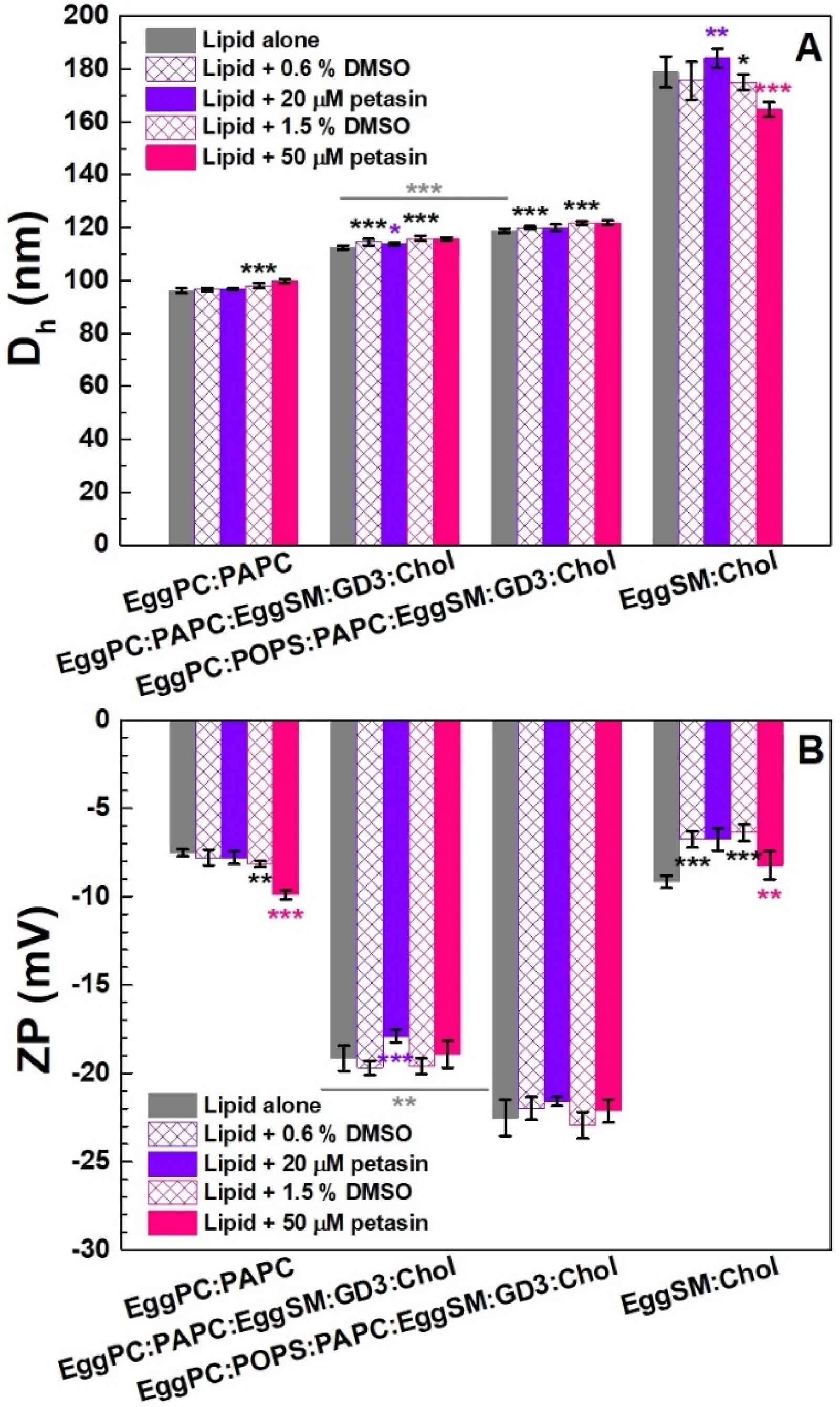
(**A**) Hydrodynamic diameter (D_h_) and (**B**) zeta potential (ZP) of reference samples (lipid alone), DMSO controls, and petasin-treated samples at 37 °C. Petasin was applied at 20 μM (violet; lipid-to-petasin molar ratio 10:1) and 50 μM (pink; lipid-to-petasin molar ratio 4:1) and compared with the corresponding DMSO controls (0.6% and 1.5% DMSO, respectively). Results are presented as mean ± SD obtained from 3 independent samples with 6 measurements per sample (*n* = 18) for D_h_ and 3 independent samples with 2 measurements per sample (*n* = 6) for ZP. Pair-Sample *t*-Test was used to assess statistical significance (* *p* < 0.05, ** *p* < 0.01, *** *p* < 0.001). Gray asterisks indicate significant differences between POPS-containing and POPS-free reference membranes. Black asterisks indicate significant differences between reference samples (gray columns) and DMSO controls (sparse-pattern columns), whereas violet and pink asterisks indicate significant differences between petasin-treated samples (solid-pattern columns) and their corresponding DMSO controls (sparse-pattern columns) at 20 and 50 μM petasin, respectively.

The effects of petasin on vesicle size, size distribution, and surface electrostatic properties are shown in Figure 9, whereas the corresponding hydrodynamic diameter (D_h_), polydispersity index (PDI), and zeta potential (ZP) values are summarized in Table 4. Relative and normalized changes in D_h_ upon petasin treatment are presented in Figure S4. Representative DLS intensity-weighted size distribution profiles are shown in Figure S5. The size distribution profiles were comparable among reference, DMSO control, and petasin-treated samples, with no additional peaks or marked peak broadening. Relative and normalized changes in ZP are presented in Figure S6. Overall, petasin produced only minor changes in D_h_, PDI, and ZP across the investigated membrane systems.

In the EggPC:PAPC membrane, petasin produced a small concentration-dependent increase in D_h_, reaching approximately 1–2% at 50 μM, whereas PDI values remained comparable with the corresponding DMSO controls. The normalized D_h_ values are presented in Figure S4. Similar responses were observed in both biomimetic mast cell membrane models. Petasin produced only minor changes in vesicle diameter, and no significant differences were detected between the POPS-containing and POPS-free membrane models at either concentration. The largest changes in D_h_ were observed in the EggSM:Chol membrane. Treatment with 50 μM petasin decreased D_h_ relative to the corresponding DMSO control and was accompanied by lower PDI values (Table 4). The normalized D_h_ values showed a concentration-dependent response in the EggSM:Chol membrane (Figure S4).

Petasin also altered the surface electrostatic properties of LUVs, as shown by zeta potential measurements (Figure 9B, Table 4, and Figure S6). In the EggPC:PAPC membrane, petasin produced a concentration-dependent decrease in ZP, with the largest change observed at 50 μM. Comparable alterations were observed in the membrane models exhibiting L_o_/L_d_ phase coexistence. The magnitude of the response differed between the POPS-free and POPS-containing membrane models. The POPS-containing membrane model exhibited the most negative reference ZP values. Petasin produced additional changes in ZP relative to the corresponding DMSO controls. The normalized ZP values differed significantly between the POPS-containing and POPS-free membrane models at 20 μM petasin but not at 50 μM (Figure S6). The change in ZP following petasin treatment in the EggSM:Chol membrane was comparable to that observed in the EggPC:PAPC membrane.

Overall, the results indicate that petasin modulates multiple membrane properties, with effects depending on lipid phase and membrane composition (Table 5). The most pronounced changes in lipid order and estimated nanodomain radius suggest alterations in membrane organization, while the decrease in dipole potential indicates changes in the membrane electrostatic environment. The largely unchanged hydrodynamic diameter suggests that these effects were not accompanied by substantial changes in vesicle size. Collectively, these findings highlight the importance of membrane composition in determining the biophysical response to petasin, although their functional implications for mast cell activity remain to be established.

## 3. Discussion

### 3.1. Molecular Basis of Petasin-Membrane Interactions, Depth Profile, and Phase Preference

The combined biophysical dataset, comprising Laurdan generalized polarization (GP), DPH anisotropy (r_DPH_), TEMPO-mediated DPH fluorescence quenching, di-8-ANEPPS dipole potential measurements, and dynamic light scattering (DLS), provides converging information on bilayer organization across multiple structural levels. Rather than indicating localized perturbation restricted to a single membrane region, the overall response suggests coordinated structural reorganization involving the membrane interface, hydrophobic core, lateral lipid organization, and membrane electrostatics. These techniques do not directly determine the molecular localization, orientation, or phase partitioning of petasin within the bilayer. Nevertheless, the responses observed across complementary biophysical parameters are consistent with petasin interacting with the lipid matrix at more than one structural level. Previous biophysical studies show that the distribution of small lipophilic molecules in phospholipid bilayers is dynamic and depends on molecular size, polarity, hydrogen-bonding capacity, and membrane composition [17,18,19,20,21]. The phase-dependent responses observed here are therefore consistent with differences in accessibility and packing between liquid-disordered and liquid-ordered environments. The dense packing of the liquid-ordered phase, stabilized by sphingomyelin and cholesterol, may present a greater energetic barrier to incorporation of bulky hydrophobic molecules than the more loosely packed liquid-disordered phase [22,23,24,25]. A possible interfacial contribution is also compatible with the Laurdan and dipole-potential responses, but the present data do not establish a specific ‘molecular wedge’ orientation or direct changes in interfacial hydration.

Our findings can be compared with previous studies of other membrane-active terpenoids. For example, nerolidol has been shown to alter phospholipid dynamics and promote lipid extraction from model membranes, while β-caryophyllene and β-caryophyllene oxide modify the thermotropic behavior of phospholipid bilayers [26,27]. Although these compounds differ structurally from petasin, their reported effects provide a relevant context for interpreting the changes observed here and support the possibility that petasin also modifies the physicochemical organization of lipid bilayers. Nevertheless, differences in molecular structure, lipid composition, and experimental conditions preclude direct equivalence between their membrane effects.

Evidence from other sesquiterpene esters further illustrates the structural dependence of these interactions. Abramov et al. [28] demonstrated that certain sesquiterpene esters increase cation permeability across lipid bilayers, whereas such effects are not necessarily shared by all related compounds. This distinction is important when interpreting the present results: changes in membrane order or dipole potential do not, by themselves, demonstrate altered membrane permeability or ion transport. Rather, the current findings establish that petasin modifies several measurable biophysical properties of the model membranes, while the functional consequences of these changes remain to be determined.

Studies of flavonoids provide an additional, complementary perspective on how the physicochemical characteristics of natural compounds influence their interactions with lipid bilayers. Arora et al. [29] reported changes in membrane fluidity following flavonoid and isoflavonoid incorporation, while Scheidt et al. [30] showed that flavonoid polarity influences molecular distribution within the membrane. Although flavonoids are structurally distinct from sesquiterpenes, these findings highlight the importance of molecular structure in determining membrane-associated effects. In the case of petasin, the complementary fluorescence-based measurements and dipole-potential analysis reveal changes in several aspects of membrane organization. However, they do not directly establish its precise location or orientation within the bilayer.

The differences observed between the liquid-disordered and liquid-ordered membrane models further suggest that the effects of petasin depend on the lipid environment. This interpretation is consistent with the heterogeneous organization of biological membranes described by Ingólfsson et al. [20], whose simulations demonstrated non-ideal lipid mixing and the formation of transient domains with liquid-ordered characteristics. Such membrane heterogeneity provides a useful framework for interpreting the phase-dependent responses observed here, although it does not establish the partitioning behavior of petasin itself.

Our previous investigations have shown that the plant-derived polyphenol compound resveratrol induces lipid membrane reorganization in a manner dependent on fatty acid unsaturation, lipid composition, and compound concentration. Resveratrol can either increase or decrease lipid order while simultaneously modulating the formation and size of L_o_ domains. These effects have been attributed to its possible spacer- or filler-like positioning within the lipid bilayer [31]. Resveratrol therefore offers a complementary perspective on the membrane-modifying effects of natural bioactive compounds, suggesting that its interactions with lipids may influence membrane-associated functions, including protein distribution, sorting, and intracellular trafficking.

Overall, the present observations extend the biophysical characterization of petasin by demonstrating changes in multiple membrane-associated parameters and by identifying differences between membrane models. However, further studies using native cellular models are needed to determine how these membrane effects contribute to petasin’s reported anti-allergic activity.

### 3.2. Modulation of Membrane Nanodomain Organization and Phase Separation

Beyond its effects on local lipid packing, DPH-TEMPO fluorescence quenching indicates that petasin also alters the lateral organization of membranes exhibiting L_o_/L_d_ phase coexistence. Because TEMPO preferentially partitions into fluid L_d_ regions, this approach provides an indirect assessment of membrane lateral heterogeneity through changes in probe accessibility [32]. The calculated nanodomain radii should therefore be regarded as approximate estimates rather than direct measurements of domain dimensions [33]. Nevertheless, the concentration-dependent changes relative to the corresponding DMSO controls are consistent with petasin-induced remodeling of membrane organization at the nanometer scale. Importantly, the observed increase in estimated nanodomain size cannot be directly attributed to a decrease in line tension. In phase-separated membranes, domain size reflects a balance between competing energetic contributions. Line tension favors minimization of the total L_o_/L_d_ interfacial perimeter and can therefore promote coalescence of smaller domains into larger ones, whereas long-range dipolar repulsion can oppose coalescence and limit domain growth. Accordingly, changes in either contribution may alter the equilibrium domain size in L_o_/L_d_ phase-separated membranes [34]. Because neither line tension nor phase-specific dipole density was measured directly in the present study, the energetic mechanism underlying the DPH-TEMPO response cannot be assigned with certainty.

The influence of membrane composition further emphasizes the importance of the lipid environment in determining the membrane response to petasin. Phosphatidylserine is known to alter membrane electrostatics, intermolecular lipid interactions, and the physicochemical environment experienced by membrane-associated proteins [3,35]. Notably, DMSO itself produced a substantial increase in the estimated nanodomain radius in the POPS-containing model. Petasin nevertheless produced an additional concentration-dependent increase relative to the corresponding DMSO controls. The magnitude of the petasin-specific increment should therefore be distinguished from the solvent effect.

Together with the Laurdan GP and DPH anisotropy data, these findings indicate that petasin affects not only local lipid packing but also the lateral organization of heterogeneous membranes. Comparable behavior has been reported for other membrane-active phytochemicals, which reorganize the physicochemical balance between coexisting membrane phases without causing membrane destabilization [10,36,37]. Because membrane nanodomains regulate the transient partitioning and interactions of signaling proteins, changes in their physicochemical properties may indirectly influence membrane-associated signaling without requiring direct interactions with individual receptors [38,39,40]. The present data do not distinguish between changes in nanodomain stability, number, lifetime, or dynamics. They support the more limited conclusion that petasin alters DPH-TEMPO accessibility in a manner consistent with changes in the estimated size and lateral organization of ordered nanodomains.

### 3.3. Remodeling of Membrane Interfacial Electrostatics and Dipole Potential

To further investigate the effects of petasin on membrane organization, membrane dipole potential (Ψ_d_) was assessed using the potentiometric probe di-8-ANEPPS. Membrane dipole potential arises from the collective orientation of phospholipid headgroups, carbonyl dipoles, and interfacial water molecules at the lipid–water interface, generating a strong electric field within the membrane [41,42]. Because Ψ_d_ is highly sensitive to changes in interfacial lipid organization and hydration, it provides complementary information to that obtained from Laurdan GP and DPH fluorescence anisotropy measurements. Alterations in membrane dipole potential have been reported following the incorporation of numerous membrane-active amphiphilic molecules into lipid bilayers and are widely regarded as sensitive indicators of changes in membrane interfacial organization [17,43].

The reduction in Ψ_d_ observed following petasin treatment indicates that its effects extend beyond changes in lipid packing to include modulation of membrane architecture of the interfacial environment. Although the molecular basis of this response cannot be resolved from the present experiments, the observed decrease in Ψ_d_ is consistent with changes in the collective organization of lipid headgroups, carbonyl dipoles, and interfacial water molecules accompanying petasin partitioning into the bilayer. This interpretation is supported by previous NMR studies demonstrating that incorporation of small lipophilic molecules can modify phospholipid headgroup orientation together with glycerol backbone dynamics, thereby altering the electrostatic properties of the membrane interface [18,19,42,43].

The phase-dependent changes in Ψ_d_ are broadly consistent with the responses observed using Laurdan GP, DPH fluorescence anisotropy, and DPH-TEMPO fluorescence quenching, indicating that the membrane response to petasin depends on the initial lipid phase state and composition. Of particular interest, the concentration-dependent decrease in average membrane dipole potential occurred in parallel with an increase in the estimated nanodomain size in the heterogeneous mast cell membrane models. Theoretical descriptions of phase-separated lipid bilayers indicate that domain size can be governed by competition between line tension, which favors domain growth, and dipole–dipole repulsion, which can oppose domain coalescence and stabilize smaller domains [34]. Thus, a reduction in the electrostatic contribution to domain repulsion could, in principle, be compatible with larger domains. However, di-8-ANEPPS reports the average membrane dipole potential, whereas the relevant energetic term depends on the difference in dipole potential or dipole density between coexisting phases. The present measurements therefore do not establish that petasin reduces L_o_/L_d_ dipolar repulsion, and the parallel changes in Ψ_d_ and estimated nanodomain size should be regarded as a mechanistically plausible association rather than evidence of causality.

Changes in membrane dipole potential may also have broader functional implications because Ψ_d_ contributes to the local electrostatic environment regulating the activity of numerous membrane-associated proteins, including ion channels, transporters, and enzymes [35,42,43,44]. Consequently, modulation of membrane dipole potential represents an additional mechanism through which petasin may regulate membrane-associated signaling independently of direct interactions with specific protein targets. Collectively, our results support a model in which petasin remodels multiple physicochemical properties of the membrane that may contribute to its reported anti-inflammatory and anti-allergic effects.

### 3.4. Preservation of Vesicular Integrity and Macrostructural Membrane Properties

To determine whether the nanoscale changes detected by fluorescence spectroscopy were accompanied by major alterations in the overall size distribution of the lipid assemblies, hydrodynamic diameter (D_h_), polydispersity index (PDI), and ζ-potential (ZP) were measured using dynamic light scattering (DLS). Representative intensity-weighted size distribution profiles remained narrow, symmetric, and monomodal in all membrane systems following both DMSO and petasin treatment, with no additional peaks or marked broadening.

Although only minor changes in vesicle size were detected in most systems, the EggSM:Chol membrane showed a reduction in D_h_ and PDI at the highest petasin concentration. This response may reflect a modest change in vesicle size distribution or homogeneity in the highly ordered membrane system. Its structural basis cannot be resolved from DLS measurements alone and therefore should not be interpreted as evidence for a specific bilayer rearrangement.

Measurements of ZP further indicate that petasin affects membrane interfacial properties in a lipid composition-dependent manner. Because petasin is electrically neutral under the experimental conditions, the observed changes are unlikely to arise from direct electrostatic interactions. Instead, they are in agreement with indirect alterations in the membrane interfacial environment, including changes in lipid headgroup orientation and hydration-layer organization, both of which are known to affect the measured ZP [45,46]. The comparatively small response of the heterogeneous mast cell membrane models further suggests that membrane surface electrostatics are determined primarily by intrinsic lipid composition, with the effects of petasin superimposed on this complex physicochemical environment.

Collectively, the DLS measurements support a membrane-remodeling effect at the level of lipid organization but, in the absence of leakage or structural imaging assays, do not by themselves demonstrate preservation of bilayer integrity.

### 3.5. Influence of POPS on Membrane Organization and Responsiveness to Petasin

Comparison of the two biomimetic mast cell membrane models highlights the contribution of POPS to the initial physicochemical properties of the membrane and to its subsequent response to petasin. Incorporation of POPS into the activated membrane model resulted in lower membrane order and a greater contribution of disordered lipid environments, which aligns with the established effects of phosphatidylserine on membrane electrostatics, lipid packing, and domain organization [3,35]. Although the present experiments do not directly assess lipid partitioning, these findings indicate that relatively small differences in membrane composition are sufficient to alter bilayer organization.

Despite these intrinsic differences, petasin produced qualitatively similar phase-dependent effects in both biomimetic mast cell membrane models. For lipid order measured by Laurdan and DPH, the petasin-induced changes did not differ significantly between the resting and activated models at either concentration. Composition-dependent differences were more evident for lateral nanodomain organization and membrane dipole potential, particularly at the higher petasin concentration. Thus, the data support an influence of membrane composition on selected aspects of the response rather than a uniformly greater sensitivity of the POPS-containing model.

The differences observed in ZP further support the conclusion that membrane composition modulates the electrostatic response to petasin. Because membrane surface charge in the activated membrane model reflects the combined contributions of phosphatidylserine, gangliosides, cholesterol, and other phospholipids, the comparatively small petasin-induced changes indicate that intrinsic lipid composition remains the primary determinant of membrane surface electrostatics. Nevertheless, the persistence of petasin-induced effects in both membrane models indicates that its membrane activity cannot be attributed to interactions with a single lipid species.

**Figure 10:**
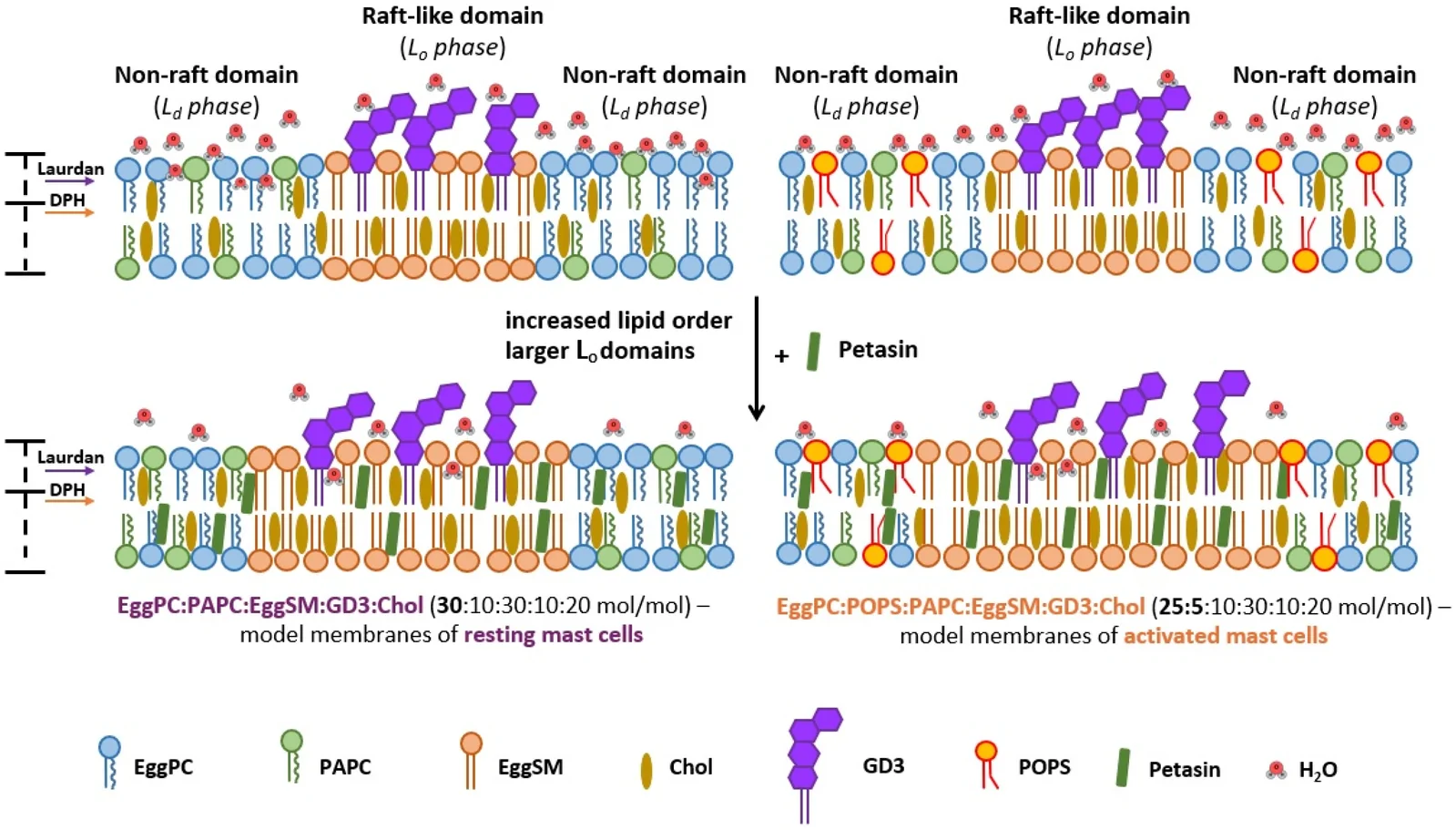
Proposed interpretation of petasin-associated changes in lipid organization and ordered nanodomain architecture in heterogeneous biomimetic membrane models of resting and activated mast cells exhibiting L_o_/L_d_ phase coexistence. The increase in lipid order was most pronounced in less ordered (L_d_) membrane environments, whereas membranes dominated by the liquid-ordered (L_o_) phase exhibited comparatively modest responses. DPH-TEMPO quenching measurements further revealed a concentration-dependent increase in the estimated dimensions of ordered nanodomains relative to the corresponding DMSO controls.

Collectively, these findings indicate that membrane composition contributes to the biophysical response to petasin, although the magnitude of this contribution differs among the measured parameters. This observation is relevant to mast cell activation, during which the plasma membrane undergoes dynamic changes in lipid distribution and lateral organization. Because the present models differ not only in POPS content but also in overall phospholipid composition, the observed differences cannot be attributed exclusively to phosphatidylserine. Their physiological significance also remains to be established in living cells. A schematic representation of the petasin-associated membrane changes observed in the resting and activated mast cell membrane models is presented in Figure 10.

### 3.6. Biological Relevance for Mast Cell Signaling

Although the ability of lipophilic compounds to modify membrane physical properties is well documented, the biological significance of these effects extends beyond changes in lipid organization alone. Studies investigating the mechanisms of action of bioactive compounds often focus primarily on their direct interactions with membrane proteins, receptors, and downstream signaling pathways, whereas the contribution of the surrounding lipid environment receives comparatively less attention. However, biological membranes are not merely passive matrices accommodating membrane proteins. Their lipid organization and physicochemical properties play an essential role in regulating protein conformation, dynamics, interactions, and activity. Consequently, compounds that modify membrane lipid organization may indirectly modulate receptor function and membrane-associated signaling pathways, potentially influencing cellular responses without necessarily interacting directly with specific protein targets.

In this context, the present findings suggest that alterations in membrane lipid organization may represent an additional membrane-mediated mechanism contributing to the biological activity of petasin. This possibility is particularly relevant to mast cells, where cholesterol- and sphingolipid-rich membrane nanodomains contribute to the spatial organization, and dynamic interactions of FcεRI, and associated signaling proteins, thereby potentially influencing mast cell responses [6,40]. Thus, the observed changes in lipid order, lateral membrane organization, and membrane dipole potential may modify the physicochemical environment in which receptor-mediated signaling occurs. Although the present study does not directly examine receptor activity or downstream signaling pathways, these findings provide a basis for further investigating the potential contribution of membrane-mediated mechanisms to the anti-inflammatory and anti-allergic effects of petasin.

Importantly, the effects of petasin extend beyond local changes in lipid packing and include alterations in the lateral organization of heterogeneous membranes, as indicated by changes in the size of ordered lipid nanodomains. The organization of coexisting lipid domains is governed by the balance between line tension at domain boundaries and dipolar interactions. Petasin-induced changes in lipid packing and interfacial electrostatics could potentially modify this balance, thereby influencing domain organization.

Furthermore, the observed reduction in membrane dipole potential points to an additional mechanism by which petasin may govern membrane-associated signaling. Because membrane dipole potential contributes to the local electric field experienced by membrane proteins, changes in Ψ_d_ may alter the energetic landscape governing conformational transitions of ion channels, transporters, and other membrane-associated proteins [42,44,47]. Although these functional consequences remain to be demonstrated experimentally, they provide a plausible biophysical framework linking membrane remodeling to cellular signaling. The present study does not directly address receptor organization or membrane protein dynamics. The observed membrane changes induced by petasin are compatible with changes in the membrane environment that can influence protein partitioning, diffusion, and intermolecular interactions [38,40]. Rather than supporting a single molecular target, these findings are consistent with a possible membrane-mediated mechanism that may complement the previously reported inhibition of leukotriene synthesis and other anti-inflammatory effects of petasin [8,48].

Taken together, these findings suggest that modulation of membrane physicochemical properties may represent one aspect of petasin’s biological activity. However, whether the observed changes occur in living mast cells and influence FcεRI signaling, degranulation, or inflammatory mediator release remains to be established experimentally. Thus, the present study provides a biophysical basis for further investigating a potential membrane-mediated contribution to the anti-inflammatory and anti-allergic effects of petasin, rather than demonstrating such a mechanism. Future research could also explore whether petasin affects additional membrane properties not assessed here, including stored curvature elastic energy, which may be influenced by changes in lipid packing and membrane organization. Such effects remain hypothetical and warrant direct investigation.

Several limitations of the present study should be acknowledged. First, the experiments were performed using simplified biomimetic membrane models that do not reproduce the full molecular complexity, leaflet asymmetry, membrane proteins, or cytoskeletal interactions of mast cell plasma membranes. Second, the fluorescence approaches provide complementary but indirect information on membrane organization and do not directly determine the molecular localization, orientation, or phase partitioning of petasin. The nanodomain radii derived from DPH-TEMPO quenching are model-based estimates, and the method does not independently resolve domain number, lifetime, or stability. Third, di-8-ANEPPS reports an average membrane dipole potential and does not resolve the phase-specific dipole potential or dipole density difference between coexisting L_o_ and L_d_ regions. Consequently, the proposed relationship between changes in dipole potential and nanodomain size remains hypothetical. Fourth, DLS excludes major aggregation or size distribution changes but does not directly demonstrate bilayer integrity or permeability. Finally, the proposed implications for FcεRI signaling remain indirect and require validation in living mast cells. Future studies combining phase-resolved biophysical measurements, molecular dynamics simulations, live-cell imaging, and functional analyses of mast cell activation will be important for establishing the physiological relevance of petasin-induced membrane remodeling.

## 4. Materials and Methods

### 4.1. Materials

The lipids used in our experiments were 1-palmitoyl-2-arachidonoyl-*sn*-glycero-3-phosphocholine (PAPC, 16:0–20:4 PC), 1-palmitoyl-2-oleoyl-*sn*-glycero-3-phospho-L-serine (POPS, 16:0–18:1 PS), L-α-phosphatidylcholine: Egg, Chicken (EggPC, with N-acyl fatty acid composition, in mol%: 14:0 (0.2%), 16:0 (32.7%), 16:1 (1.1%), 18:0 (12.3%), 18:1 (32%), 18:2 (17.1%), 20:2 (0.2%), 20:3 (0.3%), 20:4 (2.7%), 22:6 (0.4%), and unknown (1%), assayed by the supplier), sphingomyelin: Egg, Chicken (EggSM, with N-acyl fatty acid composition, in mol%: 16:0 (86%), 18:0 (6%), 22:0 (3%), 24:1 (3%), and unknown (2%), assayed by the supplier), disialoganglioside GD3: Bovine Milk (GD3) and cholesterol (Chol). All these lipids were purchased from Avanti Research (Alabaster, AL, USA) and used without further purification. The fluorescent probes 6-dodecanoyl-N, N-dimethyl-2-naphthylamine (Laurdan), 1,6-diphenyl-1,3,5-hexatriene (DPH), the radical marker 2,2,6,6-tetramethylpiperidine 1-oxyl (TEMPO) and the solvent dimethyl sulfoxide (DMSO) were ordered from Sigma-Aldrich (Darmstadt, Germany). The probe 4-(2-(6-(Dioctylamino)-2-naphthalenyl) ethenyl)-1-(3-sulfopropyl) pyridinium inner salt (di-8-ANEPPS) and Dulbecco’s phosphate-buffered saline (DPBS, no calcium, no magnesium) were purchased from ThermoFisher Scientific (Life Technologies Corporation, Waltham, MA, USA). Petasin (purity ≥ 95%) was obtained from Carl Roth (Karlsruhe, Germany).

### 4.2. Methods

#### 4.2.1. Liposome Preparation and Treatment with Petasin

Large unilamellar vesicles (LUVs) were prepared by using the extrusion method (LUVET). Lipids and fluorescent probes (Laurdan, DPH, di-8-ANEPPS) were mixed at the required molar ratios using stock solutions prepared in chloroform or chloroform:methanol 9:1 (*v*/*v*) for lipids and Laurdan, tetrahydrofuran (THF) for DPH, and ethanol for di-8-ANEPPS. Organic solvents were removed under a gentle stream of nitrogen, followed by overnight vacuum desiccation. The resulting lipid films were hydrated with DPBS (1×, pH 7.4, containing 150 mM NaCl) and subjected to three cycles of heating (60 °C, 5 min), vortex mixing (1 min), ultrasonication (1 min), and cooling (5 min). The resulting multilamellar vesicles (MLVs) were extruded at 60 °C using an Avanti Mini-Extruder (Alabaster, AL, USA) through 800-nm (11 passes) and 100-nm (21 passes) Nuclepore™ polycarbonate track-etched membranes (Whatman™, Cytiva, Marlborough, MA, USA) to produce homogeneous LUVs [49,50]. *Only the EggPC:PAPC LUVs representing the pure L_d_ phase were prepared at room temperature using a modified hydration protocol without heating or cooling. As reported previously [51], these highly fluid lipids remain in the liquid-disordered state under ambient conditions, whereas additional thermal treatment could alter membrane organization and potentially affect the measured biophysical parameters.

A 5 mg/mL stock solution of petasin was prepared in chloroform:methanol (1:1, *v*/*v*). After evaporation of the organic solvents, the residue was reconstituted in DMSO:H_2_O (9:1, *v*/*v*) to obtain a working solution containing 1 mg/mL petasin. The petasin solution and the corresponding DMSO-H_2_O mixture were added to the LUV suspensions by replacing an equivalent volume of DPBS to distinguish solvent-induced effects from those specifically attributable to petasin. Accordingly, three experimental groups were prepared: (i) reference LUVs suspended in DPBS, (ii) controls containing DMSO at final concentrations of 0.6% or 1.5%, and (iii) petasin-treated LUVs containing 20 or 50 μM petasin. The concentrations of petasin were selected based on the study by [52], in which petasin was investigated in Vero E6 cells at concentrations up to 63.2 μM. At a constant lipid concentration of 200 μM, these concentrations correspond to lipid-to-petasin molar ratios of 10:1 and 4:1, respectively. The two concentrations were used to compare petasin-induced changes in membrane biophysical properties at different exposure levels. After incubation for 4 h at 37 °C, all measurements were performed on the same day.

#### 4.2.2. Fluorescence Spectroscopy of Laurdan- and DPH-Labeled LUVs

Laurdan fluorescence spectroscopy and DPH steady-state fluorescence anisotropy were used to assess membrane order at different depths of the lipid bilayer. Laurdan reports on lipid order at the hydrophobic–hydrophilic interface, whereas DPH probes lipid packing within the hydrophobic core of the acyl-chain region.

Laurdan is a polarity-sensitive naphthalene fluorophore that partitions below the phospholipid glycerol backbone, in the vicinity of the *sn-1* carbonyl groups [53], in a region characterized by limited water penetration. Upon excitation, its dipole moment increases, inducing dipolar relaxation of surrounding water molecules [54]. The resulting emission spectrum depends on membrane order and interfacial hydration, exhibiting a red-shifted maximum (~490 nm) in disordered membranes and a blue-shifted maximum (~440 nm) in ordered membranes. These spectral changes were quantified using the generalized polarization (GP) parameter [55]:

GP = (I_440_ − I_490_)/(I_440_ + I_490_), (1)

where I_440_ and I_490_ correspond to emission intensities at 440 and 490 nm. GP values range from −1 (highly disordered membranes) to +1 (highly ordered membranes) [56]. Fluorescence emission spectra were recorded using excitation at 355 nm over the wavelength range 390–600 nm with a Tecan Infinite 200 PRO microplate reader (Tecan Group Ltd., Männedorf, Switzerland) using Greiner Bio-One F-bottom 96-well plates. Lipid:Laurdan mixtures were prepared at a 200:1 molar ratio.

DPH is a non-polar rod-like polyene whose absorption and emission transition dipoles are aligned along its molecular axis [57,58]. Its fluorescence anisotropy depends on rotational mobility, which is restricted by interactions with surrounding lipid acyl chains [59]. Steady-state fluorescence anisotropy therefore provides a measure of acyl-chain packing within the membrane hydrophobic core. Measurements were performed using an FP-8300 spectrofluorometer (Jasco, Heckmondwike, West Yorkshire, UK) equipped with polarizers, a xenon arc lamp, a thermostated cuvette holder, and a quartz cuvette. Fluorescence anisotropy (r_DPH_) was calculated as [60]:

r_DPH_ = (I_VV_ − GI_VH_)/(I_VV_ + 2GI_VH_) (2)

where G = I_HV_/I_HH_ is the instrumental correction factor determined individually for each sample. I_VV_, I_VH_, I_HV_ and I_HH_ denote the measured fluorescence intensities for the corresponding excitation/emission polarization combinations. Higher r_DPH_ values indicate restricted probe rotation and greater membrane order, whereas lower values reflect increased rotational mobility and membrane fluidity. Lipid:DPH samples were prepared at a molar ratio of 200:1. Excitation was set at 358 nm, fluorescence emission was monitored at 430 nm, and both excitation and emission slit widths were set to 5 nm.

#### 4.2.3. DPH-TEMPO Fluorescence Quenching

DPH fluorescence quenching by the water-soluble nitroxide radical TEMPO was used to assess membrane lateral heterogeneity and estimate nanodomain size in LUVs in the absence and presence of petasin. TEMPO preferentially partitions into liquid-disordered (L_d_) regions, whereas DPH distributes throughout both liquid-disordered (L_d_) and liquid-ordered (L_o_) membrane phases. Consequently, DPH fluorescence is quenched more efficiently in disordered membrane regions, whereas ordered domains are less accessible to TEMPO [61,62,63]. TEMPO quenches DPH fluorescence through short-range collisional interactions, resulting in decreased fluorescence intensity and excited-state lifetime of the fluorophore [64]. The remaining DPH fluorescence in the presence of TEMPO was expressed as follows:

Q = F/F_0_ (3)

where F_0_ and F denote the fluorescence intensities of DPH in the absence and presence of TEMPO, respectively. Higher Q values indicate reduced quenching, consistent with greater membrane order and/or a larger contribution of ordered membrane domains, whereas lower Q values indicate increased probe accessibility within disordered membrane regions. Measurements were performed at 37 °C using a Tecan Infinite 200 PRO microplate reader (Tecan Group Ltd., Männedorf, Switzerland) with Greiner Bio-One F-bottom 96-well plates. Lipid:DPH mixtures were prepared at a 1000:1 molar ratio. DPH fluorescence was recorded using excitation and emission wavelengths of 358 and 430 nm, respectively [65]. Samples (final lipid concentration 200 μM) were incubated for 10 min before recording baseline fluorescence (F_0_). TEMPO was then added to a final concentration of 2 mM (ethanolic stock solution), followed by an additional 10 min incubation before measuring quenched fluorescence (F). Three independent samples were analyzed, each measured 15 times (*n* = 45).

For membranes exhibiting L_o_/L_d_ phase coexistence, the apparent nanodomain radius (R_dom_, Å) was estimated from the measured Q values according to the restricted-diffusion model proposed by [63]:

R_dom_ = R_o_ × ((Q_X_ − Q_EggPC:PAPC_)/(Q_SM:Chol_ − Q_EggPC:PAPC_)) (4)

where Q_X_ represents the Q value of the biomimetic system exhibiting L_o_/L_d_ phase coexistence in resting or activated mast cells, R_o_ (≈48 Å; approximately four times the effective quenching radius, R_c_) represents the characteristic distance for nearly 50% DPH quenching, whereas R_c_ (≈12 Å) corresponds to the effective quenching radius of TEMPO [33,66]. Q_EggPC:PAPC_ and Q_SM:Chol_ correspond to the reference quenching values of the pure L_d_ and L_o_ membrane models, respectively. These reference values define the lower and upper limits of quenching and were used to estimate the apparent nanodomain radii of membranes exhibiting L_o_/L_d_ phase coexistence.

#### 4.2.4. Fluorescence Spectroscopy of di-8-ANEPPS-Labeled LUVs for Assessment of Membrane Dipole Potential

Membrane dipole potential (Ψ_d_) was determined in di-8-ANEPPS-labeled LUVs in the absence and presence of petasin. The voltage-sensitive styryl probe di-8-ANEPPS partitions into the outer leaflet of lipid bilayers and reports changes in the interfacial electric field generated by the collective orientation of lipid headgroups, carbonyl dipoles, and interfacial water molecules. Consequently, changes in the excitation spectrum reflect variations in membrane dipole potential associated with alterations in membrane composition and lipid organization [41,42,43]. Membrane dipole potential was assessed using the excitation-ratiometric method. The excitation ratio (R_ex_) was calculated as follows:

R_ex_ = I_420_/I_520_ (5)

where I_420_ and I_520_ represent fluorescence intensities recorded at 670 nm following excitation at 420 and 520 nm. Higher R_ex_ values correspond to higher Ψ_d_. For quantitative comparison, Ψ_d_ was calculated from R_ex_ using the empirical calibration equation:

Ψ_d_ = (R_ex_ + 0.3)/0.0043 (6)

where the empirical constants (0.3 and 0.0043) were adopted from previously established correlations between di-8-ANEPPS excitation ratios and membrane dipole potentials determined in model lipid membranes [41,42,67]. Changes in R_ex_ and the corresponding Ψ_d_ values were used to evaluate the effects of petasin on membrane dipole potential. Measurements were performed at 37 °C using a Tecan Infinite 200 PRO microplate reader (Tecan Group Ltd., Männedorf, Switzerland) with Greiner Bio-One F-bottom 96-well plates. Lipid:di-8-ANEPPS mixtures were prepared at a 250:1 molar ratio. 

#### 4.2.5. Vesicle Size and ζ Potential Measurements

Hydrodynamic diameter (D_h_), polydispersity index (PDI), and ζ potential (ZP) of LUVs were determined at 37 °C using a Zetasizer Nano ZS (Malvern Instruments, Malvern, UK) equipped with a 4 mW He–Ne laser (633 nm) and a temperature-controlled cuvette holder. For each measurement, 1 mL of sample (final lipid concentration 200 μM) was transferred to a disposable folded capillary cell fitted with a ZEN1002 dip cell (Malvern Instruments) containing palladium electrodes for electrophoretic measurements. Samples were equilibrated at 37 °C for 10 min before analysis.

Hydrodynamic diameter and PDI were determined by dynamic light scattering (DLS) from fluctuations in scattered light intensity arising from Brownian motion of vesicles in suspension. The translational diffusion coefficients were converted into hydrodynamic diameters using the Stokes–Einstein equation, assuming spherical particles. Particle size distributions are reported as intensity-weighted values [68].

Ζeta potential was determined by electrophoretic light scattering (ELS) using laser Doppler velocimetry combined with phase analysis light scattering (PALS). Electrophoretic mobility was converted to ZP using the Smoluchowski approximation implemented in the instrument software, which is appropriate for aqueous dispersions of moderate ionic strength [69].

Three independent samples were analyzed for each experimental condition, with multiple instrument runs per sample. Results are presented as mean ± SD.

#### 4.2.6. Calculation of Petasin Effects on LUV Membranes

Relative percentage changes (ΔX, %) were calculated with respect to the corresponding control samples in the absence of petasin. Because percentage changes depend on baseline values, small absolute differences may produce relatively large percentage changes when control values are low, whereas similar absolute differences yield smaller percentage changes when baseline values are high. Therefore, relative changes were interpreted together with the corresponding absolute values. Relative changes were calculated as follows:

ΔX (%) = ((X_petasin_ − X_control_)/X_control_) × 100 (7)

where X represents the measured parameter (Laurdan Generalized Polarization (GP), DPH anisotropy (r_DPH_), dipole potential (Ψ_d_), or hydrodynamic diameter (D_h_)), and X_petasin_ and X_control_ denote values measured in the presence and absence of petasin, respectively.

To enable direct comparison between membrane systems with different initial physicochemical properties, petasin-induced changes were additionally normalized to the experimentally defined liquid-disordered (L_d_)—liquid-ordered (L_o_) phase range (ΔX*_norm_*, %). In this approach, the difference between the corresponding pure L_d_ and L_o_ reference membranes was defined as 100%. Accordingly:

- The GP difference between EggPC:PAPC (−0.19) and EggSM:Chol (0.45) was defined as 100% (ΔGP = 0.64);
- The r_DPH_ difference between EggPC:PAPC (0.11) and EggSM:Chol (0.34) was defined as 100% (Δr_DPH_ = 0.23);
- The Ψ_d_ difference between EggPC:PAPC (340 mV) and EggSM:Chol (540 mV) was defined as 100% (ΔΨ_d_ = 200 mV);
- The D_h_ difference between EggPC:PAPC (96 nm) and EggSM:Chol (179 nm) was defined as 100% (ΔD_h_ = 83 nm).

Normalized changes were calculated as follows:

ΔX*_norm_* (%) = ((X_petasin_ − X_control_)/(X_Lo_ − X_Ld_)) × 100 (8)

where X_Lo_ and X_Ld_ correspond to the values measured in the pure L_o_ and L_d_ reference membranes, respectively. This normalization places all membrane systems on a common physicochemical scale, thereby facilitating comparison of petasin-induced effects across membranes differing in lipid composition and initial phase state.

Because ZP does not directly reflect membrane phase behavior, a different normalization procedure was applied. ZP values were normalized to the experimentally observed electrostatic range across all investigated lipid systems, with the least negative value defined as 0% and the most negative value as 100%. Normalized ZP changes were calculated as follows:

ΔZP*_norm_* (%) = ((ZP_petasin_ − ZP_control_)/(ZP_max_ − ZP_min_)) × 100 (9)

where ZP_petasin_ and ZP_control_ represent ζ potential values measured in the presence and absence of petasin, respectively, while ZP_max_ and ZP_min_ correspond to the most negative (EggPC:POPS:PAPC:EggSM:GD3:Chol 25:5:10:30:10:20) and least negative (EggPC:PAPC 75:25) values observed among all membrane systems. The experimentally accessible electrostatic window was defined by the difference between these two reference systems (−22.52 and −7.52 mV), corresponding to a total range of 15 mV, which was taken as 100% for normalization. Normalized ZP values describe the relative position of each membrane within the experimentally observed electrostatic range, whereas relative percentage changes quantify the directional effect of petasin relative to the corresponding control. Because these metrics use different reference frames, their numerical values and signs are not directly comparable.

#### 4.2.7. Statistical Analysis

Statistical analyses were performed using OriginPro 9.0 (OriginLab Corporation, Northampton, MA, USA). Differences between reference samples, DMSO-only controls, and petasin-treated samples were evaluated using a Pair-sample *t*-test. Comparisons were performed between reference samples and the corresponding DMSO controls (black asterisks), between DMSO controls and the corresponding petasin-treated samples (violet and pink asterisks), and between the two petasin concentrations, as presented in the Supplementary Data. Normality of the data was assessed using the Kolmogorov–Smirnov test. Statistical significance was defined as * *p* < 0.05, ** *p* < 0.01, *** *p* < 0.001, whereas ns indicates non-significant differences.

## 5. Conclusions

The present study demonstrates that petasin modulates the biophysical properties of biomimetic mast cell membrane models in a lipid composition- and phase-dependent manner. The combined analyses using fluorescence spectroscopy, membrane dipole potential assays, and dynamic light scattering demonstrated that petasin modulates several aspects of lipid bilayer behavior, including lipid packing, lateral organization, and interfacial electrostatic properties, while influencing vesicle physicochemical characteristics without inducing detectable aggregation or substantial changes in vesicle size distribution.

The integrated biophysical analysis indicates that the membrane response to petasin depends on the initial physicochemical state of the lipid bilayer. Petasin increases lipid order most clearly in relatively disordered membranes, exerts only minor effects on highly ordered sphingomyelin/cholesterol-rich bilayers, alters DPH-TEMPO accessibility in a manner consistent with increased estimated nanodomain size in heterogeneous membranes, and decreases membrane dipole potential. Together, these findings indicate that petasin modulates multiple physicochemical features of the membrane rather than producing a single localized perturbation.

To our knowledge, this study provides the first systematic biophysical characterization of petasin effects in biomimetic mast cell membrane models. The results support the possibility that modulation of membrane physicochemical properties represents an additional component contributing to the reported anti-inflammatory and anti-allergic activities of petasin. Rather than replacing previously identified molecular targets, membrane remodeling may complement established mechanisms by modifying the physicochemical environment in which membrane-associated signaling occurs.

Although the physiological relevance of these findings remains to be established in living cells, the present work identifies membrane organization as a potential contributor to the biological activity of petasin and provides a framework for future studies integrating membrane biophysics, molecular dynamics simulations, and functional analyses of mast cell activation.

## Figures and Tables

**Table 1:** Effect of petasin (50 μM) on membrane lipid order determined by Laurdan generalized polarization (GP), reflecting lipid order at the hydrophobic–hydrophilic interface, and DPH fluorescence anisotropy (r_DPH_), reflecting lipid order within the hydrophobic core of the membrane bilayer. Results are expressed as absolute (ΔGP and Δr_DPH_) and normalized percentage changes relative to the L_d_–L_o_ range (ΔGP*_norm_* (%), Δr_DPH *norm*_ (%)) at 37 °C. Values are presented as mean ± SD. Positive values indicate an increase in lipid order, whereas negative values indicate a decrease in lipid order.

Phase State	Lipid Composition	ΔGP	ΔGP*_norm_* (%)	Δr_DPH_	Δr_DPH *norm*_ (%)
L_d_	EggPC:PAPC	0.027 ± 0.002	4.13 ± 0.32	0.011 ± 0.004	4.73 ± 1.68
L_o_/L_d_	EggPC:PAPC:EggSM:GD3:Chol	0.010 ± 0.005	1.60 ± 0.85	0.004 ± 0.001	1.91 ± 0.53
L_o_/L_d_	EggPC:POPS:PAPC:EggSM:GD3:Chol	0.009 ± 0.006	1.41 ± 0.99	0.005 ± 0.003	2.04 ± 1.09
L_o_	EggSM:Chol	−0.003 ± 0.003	−0.46 ± 0.44	−0.002 ± 0.001	−0.93 ± 0.58

**Table 2:** DPH fluorescence quenching by TEMPO expressed as Q (F/F_0_) values and the corresponding estimated membrane nanodomain radii (R_dom_, Å) in LUVs, used as biomimetic systems exhibiting L_o_/L_d_ phase coexistence in resting and activated mast cells, at 37 °C. ΔR_dom_ (Å) represents the absolute change in nanodomain radius relative to the appropriate reference sample (DMSO controls versus lipid-alone samples; petasin-treated samples versus the corresponding DMSO controls). Results are presented as mean ± SD obtained from 3 independent samples with 15 measurements per sample (*n* = 45).

Lipid Composition	Q (F/F_0_)	R_dom_ (Å)	ΔR_dom_ (Å)
EggPC:PAPC:EggSM:GD3:Chol			
Lipid alone	0.78 ± 0.01	34.02 ± 1.25	-
Lipid + 0.6% DMSO	0.78 ± 0.01	34.42 ± 1.27	0.40 ± 1.27
Lipid + 20 μM petasin	0.80 ± 0.01	36.13 ± 0.97	1.71 ± 0.97
Lipid + 1.5% DMSO	0.78 ± 0.02	34.57 ± 1.94	0.55 ± 1.94
Lipid + 50 μM petasin	0.81 ± 0.02	37.46 ± 1.96	2.89 ± 1.96
EggPC:POPS:PAPC:EggSM:GD3:Chol			
Lipid alone	0.77 ± 0.02	32.22 ± 2.32	-
Lipid + 0.6% DMSO	0.80 ± 0.01	36.21 ± 1.61	3.99 ± 1.61
Lipid + 20 μM petasin	0.81 ± 0.02	37.43 ± 1.95	1.22 ± 1.95
Lipid + 1.5% DMSO	0.80 ± 0.02	36.60 ± 1.96	4.38 ± 1.96
Lipid + 50 μM petasin	0.83 ± 0.02	40.31 ± 1.94	3.71 ± 1.94

**Table 3:** Di-8-ANEPPS fluorescence excitation ratio (R_ex_) determined from fluorescence intensities measured at 670 nm upon excitation at 420 and 520 nm, membrane dipole potential (Ψ_d_, mV), and normalized changes in membrane dipole potential relative to the L_d_–L_o_ range (ΔΨ_d *norm*_, %) for reference samples (lipid alone), DMSO controls, and petasin-treated LUVs at 37 °C. Values are presented as mean ± SD obtained from 3 independent samples with 15 measurements per sample (*n* = 45). Positive ΔΨ_d *norm*_ values indicate an increase in membrane dipole potential, whereas negative values indicate a decrease.

Lipid Phase	Lipid Composition	R_ex_	Ψ_d_ (mV)	ΔΨ_d *norm*_ (%)
L_d_	EggPC:PAPC			
	Lipid alone	1.16 ± 0.01	340 ± 3	-
	Lipid + 0.6% DMSO	1.16 ± 0.01	340 ± 3	0.01 ± 1.53
	Lipid + 20 μM petasin	1.13 ± 0.01	333 ± 3	−3.64 ± 1.53
	Lipid + 1.5% DMSO	1.16 ± 0.02	340 ± 4	−0.15 ± 1.77
	Lipid + 50 μM petasin	1.10 ± 0.01	325 ± 3	−7.27 ± 1.29
L_o_/L_d_	EggPC:PAPC:EggSM:GD3:Chol			
	Lipid alone	1.01 ± 0.01	305 ± 3	-
	Lipid + 0.6% DMSO	1.01 ± 0.01	305 ± 3	−0.02 ± 1.25
	Lipid + 20 μM petasin	0.98 ± 0.01	297 ± 3	−3.96 ± 1.23
	Lipid + 1.5% DMSO	1.01 ± 0.01	305 ± 3	0.37 ± 1.43
	Lipid + 50 μM petasin	0.94 ± 0.01	288 ± 2	−8.55 ± 1.07
L_o_/L_d_	EggPC:POPS:PAPC:EggSM:GD3:Chol			
	Lipid alone	1.06 ± 0.01	317 ± 3	-
	Lipid + 0.6% DMSO	1.06 ± 0.01	317 ± 3	−0.08 ± 1.25
	Lipid + 20 μM petasin	1.03 ± 0.01	308 ± 3	−4.07 ± 1.40
	Lipid + 1.5% DMSO	1.07 ± 0.01	318 ± 3	0.41 ± 1.53
	Lipid + 50 μM petasin	0.99 ± 0.01	299 ± 3	−9.05 ± 1.25
L_o_	EggSM:Chol			
	Lipid alone	2.02 ± 0.04	540 ± 9	-
	Lipid + 0.6% DMSO	2.03 ± 0.03	542 ± 7	0.54 ± 3.63
	Lipid + 20 μM petasin	2.01 ± 0.03	538 ± 8	−1.81 ± 3.98
	Lipid + 1.5% DMSO	2.03 ± 0.04	541 ± 8	0.29 ± 4.00
	Lipid + 50 μM petasin	1.99 ± 0.04	532 ± 8	−4.56 ± 4.10

**Table 4:** Hydrodynamic diameter (D_h_, nm), polydispersity index (PDI), and zeta potential (ZP, mV) of reference samples (lipid alone), DMSO controls, and petasin-treated LUVs at 37 °C. Values are presented as mean ± SD.

Lipid Phase	Lipid Composition	D_h_ (nm)	PDI	ZP (mV)
L_d_	EggPC:PAPC			
	Lipid alone	96.21 ± 0.95	0.11 ± 0.02	−7.52 ± 0.21
	Lipid + 0.6% DMSO	96.69 ± 0.62	0.14 ± 0.02	−7.79 ± 0.45
	Lipid + 20 μM petasin	96.80 ± 0.31	0.15 ± 0.03	−7.80 ± 0.37
	Lipid + 1.5% DMSO	98.06 ± 0.93	0.15 ± 0.03	−8.15 ± 0.17
	Lipid + 50 μM petasin	99.69 ± 0.70	0.14 ± 0.02	−9.90 ± 0.25
L_o_/L_d_	EggPC:PAPC:EggSM:GD3:Chol			
	Lipid alone	112.47 ± 0.74	0.10 ± 0.03	−19.15 ± 0.71
	Lipid + 0.6% DMSO	114.50 ± 1.17	0.10 ± 0.04	−19.68 ± 0.40
	Lipid + 20 μM petasin	113.81 ± 0.56	0.08 ± 0.02	−17.89 ± 0.36
	Lipid + 1.5% DMSO	115.88 ± 0.96	0.10 ± 0.03	−19.59 ± 0.45
	Lipid + 50 μM petasin	115.70 ± 0.61	0.09 ± 0.02	−18.92 ± 0.76
L_o_/L_d_	EggPC:POPS:PAPC:EggSM:GD3:Chol			
	Lipid alone	118.92 ± 0.74	0.07 ± 0.03	−22.52 ± 1.03
	Lipid + 0.6% DMSO	120.07 ± 0.51	0.09 ± 0.03	−21.99 ± 0.64
	Lipid + 20 μM petasin	119.93 ± 1.24	0.08 ± 0.02	−21.59 ± 0.25
	Lipid + 1.5% DMSO	121.87 ± 0.74	0.07 ± 0.02	−22.94 ± 0.74
	Lipid + 50 μM petasin	121.94 ± 0.84	0.07 ± 0.02	−22.12 ± 0.66
L_o_	EggSM:Chol			
	Lipid alone	178.92 ± 5.87	0.28 ± 0.04	−9.15 ± 0.35
	Lipid + 0.6% DMSO	175.56 ± 7.15	0.25 ± 0.05	−6.76 ± 0.45
	Lipid + 20 μM petasin	184.19 ± 3.44	0.28 ± 0.03	−6.76 ± 0.64
	Lipid + 1.5% DMSO	175.10 ± 2.95	0.25 ± 0.02	−6.38 ± 0.47
	Lipid + 50 μM petasin	164.72 ± 2.77	0.17 ± 0.03	−8.24 ± 0.80

**Table 5:** Summary of petasin-induced changes at 50 μM in the biophysical properties of biomimetic mast cell membrane models. The table summarizes the effects of petasin on interfacial and hydrophobic core lipid order (GP and r_DPH_, respectively), estimated ordered nanodomain radius (R_dom_), membrane dipole potential (Ψ_d_), hydrodynamic diameter (D_h_), and zeta potential (ZP) in the L_d_ and L_o_ phases and in biomimetic models of resting and activated mast cell membranes exhibiting L_o_/L_d_ phase coexistence. The observed responses varied according to the membrane system, with differences in the magnitude and direction of changes across the investigated parameters. Changes described as not statistically significant should be interpreted accordingly.

Parameter	L_d_ Phase	Resting Mast cell Model (L_o_/L_d_)	Activated Mast Cell Model (L_o_/L_d_)	L_o_ Phase
Interfacial lipid order (GP)	Increased markedly	Increased moderately	Increased moderately (not significantly different from the resting model)	Decreased slightly
Hydrophobic core lipid order (r_DPH_)	Increased markedly	Increased moderately	Increased moderately (not significantly different from the resting model)	Decreased slightly
Estimated nanodomain radius (R_dom_)	N/A	Increased	Increased more than in the resting model	N/A
Membrane dipole potential (Ψ_d_)	Decreased	Decreased more than in the L_d_ phase	Decreased most strongly among the tested systems	Decreased, but less than in the other systems
Hydrodynamic diameter (D_h_)	No substantial change	No substantial change	No substantial change	Decreased
Zeta potential (ZP)	Shifted toward more negative values	Shifted toward less negative values (not statistically significant)	Shifted toward less negative values (not statistically significant)	Shifted toward more negative values

Note: Not applicable (N/A) indicates that a parameter is irrelevant to that specific membrane system. Specifically, domain size (R_dom_) applies only to heterogeneous membranes with L_o_/L_d_ phase coexistence. It is not defined for pure L_d_ or L_o_ phases, as these homogeneous systems lack the coexisting regions required to form distinct domains.

## Data Availability

The original contributions presented in this study are included in the article/Supplementary Materials. Further inquiries can be directed to the corresponding authors.

## Supplementary Materials

The following supporting information can be downloaded at: https://www.mdpi.com/article/10.3390/molecules31203766/s1, Figure S1: (**A**) Relative (ΔGP, %) and (**B**) normalized (ΔGP*_norm_*, %) changes in Laurdan GP induced by petasin in LUVs at 37 °C. Relative changes (ΔGP, %) were calculated with respect to the corresponding DMSO controls, whereas normalized values (ΔGP*_norm_*, %) were scaled to the experimentally defined L_d_–L_o_ phase range (0–100%). Results are presented as mean ± SD (*n* = 45). Statistical significances between the effects of 20 μM (violet) and 50 μM (pink) petasin are indicated black asterisks (*** *p* < 0.001). Differences between the two heterogeneous membrane models were not statistically significant (indicated by connecting lines and ns in violet and pink colour) at either petasin concentration.; Figure S2: (**A**) Relative (Δr_DPH_, %) and (**B**) normalized (Δr_DPH *norm*_, %) changes in DPH steady-state fluorescence anisotropy induced by petasin in LUVs at 37 °C. Relative changes (Δr_DPH_, %) were calculated with respect to the corresponding DMSO controls, whereas normalized values (Δr_DPH *norm*_, %) were scaled to the experimentally defined L_d_–L_o_ phase range (0–100%). Results are presented as mean ± SD (*n* = 18). Statistical significance between the effects of 20 μM (violet) and 50 μM (pink) petasin is indicated by black asterisks (* *p* < 0.05, ** *p* < 0.01, *** *p* < 0.001). Differences between the two heterogeneous membrane models were not statistically significant (indicated by connecting lines and ns in violet and pink colour) at either petasin concentration.; Figure S3: (**A**) Relative (ΔΨ_d_, %) and (**B**) normalized (ΔΨ_d *norm*_, %) changes in membrane dipole potential induced by petasin in di-8-ANEPPS-labeled LUVs at 37 °C. Relative changes (ΔΨ_d_, %) were calculated with respect to the corresponding DMSO controls, whereas normalized values (ΔΨ_d *norm*_, %) were scaled to the experimentally defined L_d_–L_o_ phase range (0–100%). Results are presented as mean ± SD (*n* = 45). Statistical significance between the effects of 20 μM (violet) and 50 μM (pink) petasin is indicated by black asterisks (*** *p* < 0.001). A statistically significant difference between the two heterogeneous membrane models was observed only at 50 μM petasin (* *p* < 0.05; indicated by connecting line in pink colour), whereas no significant difference was detected at 20 μM petasin.; Figure S4: (**A**) Relative (ΔD_h_, %) and (**B**) normalized (ΔD_h *norm*_, %) changes in hydrodynamic diameter induced by petasin in LUVs at 37 °C. Relative changes (ΔD_h_, %) were calculated with respect to the corresponding DMSO controls, whereas normalized values (ΔD_h *norm*_, %) were scaled to the experimentally defined L_d_–L_o_ phase range (0–100%). Results are presented as mean ± SD (*n* = 18). Statistical significance between the effects of 20 μM (violet) and 50 μM (pink) petasin is indicated in black colour (** *p* < 0.01, *** *p* < 0.001, ns, not significant). Differences between the two heterogeneous membrane models were not statistically significant (indicated by connecting lines and ns in violet and pink colour) at either petasin concentration.; Figure S5: Representative intensity-weighted size distribution profiles of LUVs at 37 °C following treatment with DMSO or petasin. Size distributions are presented as intensity-weighted hydrodynamic diameter (D_h_) profiles obtained by dynamic light scattering. Reference samples (lipid alone) are shown in black, DMSO controls in light violet (0.6% DMSO) and light pink (1.5% DMSO), and petasin-treated samples in dark violet (20 μM) and dark pink (50 μM). The corresponding quantitative values of D_h_ and polydispersity index (PDI) are presented in Figure 9A and Table 4.; Figure S6: Effect of petasin on the surface electrostatic properties of LUVs at 37 °C. Data are presented as (**A**) relative percentage changes in ζ-potential (ΔZP, %) with respect to the corresponding DMSO controls and (**B**) normalized percentage changes (ΔZP *_norm_*, %) scaled to the experimentally defined electrostatic window between the least negative EggPC:PAPC (75:25 mol/mol) and the most negative EggPC:POPS:PAPC:EggSM:GD3:Chol (25:5:10:30:10:20 mol/mol) reference ζ-potential values. Thus, normalized values reflect the relative position within the experimentally accessible electrostatic range. Results are presented as mean ± SD (*n* = 6). Statistical significance between the effects of 20 μM (violet) and 50 μM (pink) petasin is indicated in black colour (* *p* < 0.05, ** *p* < 0.01, *** *p* < 0.001, ns, not significant). A statistically significant difference between the two heterogeneous membrane models was observed at 20 μM petasin (violet lines and asterisks), whereas no significant difference was detected at 50 μM petasin (pink lines and asterisks).

## Abbreviations

5-LOX	5-lipoxygenase
Chol	cholesterol
D_h_	hydrodynamic diameter
di-8-ANEPPS	4-(2-(6-(Dioctylamino)-2-naphthalenyl) ethenyl)-1-(3-sulfopropyl) pyridinium inner salt
DLS	dynamic light scattering
DMSO	dimethyl sulfoxide
DPBS	Dulbecco’s phosphate-buffered saline
DPH	1,6-diphenyl-1,3,5-hexatriene
EggPC	egg phosphatidylcholine
EggSM	egg sphingomyelin
GD3	disialoganglioside
GP	generalized polarization
IgE	immunoglobulin E
Laurdan	6-dodecanoyl-N, N-dimethyl-2-naphthylamine
L_d_	liquid-disordered
L_o_	liquid-ordered
LTC_4_	leukotriene C_4_
LTD_4_	leukotriene D_4_
LTE_4_	leukotriene E_4_
LUVs	large unilamellar vesicles
PAPC	1-palmitoyl-2-arachidonoyl-*sn*-glycero-3-phosphocholine
PDI	polydispersity index
POPS	1-palmitoyl-2-oleoyl-*sn*-glycero-3-phospho-L-serine
PS	phosphatidylserine
R_dom_	nanodomain radii
r_DPH_	DPH anisotropy
R_ex_	excitation ratio
SD	standard deviation
TEMPO	2,2,6,6-tetramethylpiperidine 1-oxyl
ZP	zeta potential
Ψ_d_	dipole potential
