# Supplementary material for: Petasin at the Membrane: Fine-Tuning Lipid Organization and Interfacial Electrostatics in Mast Cell Plasma Membrane Models
Source: Molecules. 2026 Oct 20;31(20):3766. doi: 10.3390/molecules31203766 (PMC13707746; doi:10.3390/molecules31203766)
Supplement: Supplementary file 1 [file molecules-31-03766-s001.zip › molecules-4617998-supplementary.pdf]

Supplementary data

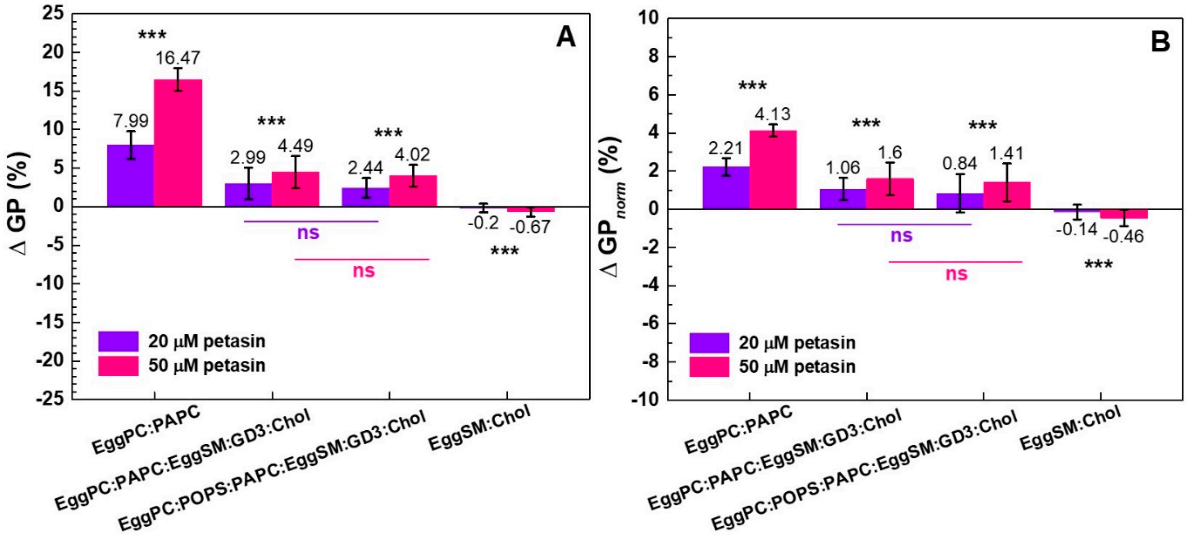

Figure S1. (**A**) Relative (ΔGP, %) and (**B**) normalized (ΔGP_{norm}, %) changes in Laurdan GP induced by petasin in LUVs at 37°C. Relative changes (ΔGP, %) were calculated with respect to the corresponding DMSO controls, whereas normalized values (ΔGP_{norm}, %) were scaled to the experimentally defined L_d–L_o phase range (0–100%). Results are presented as mean ± SD (*n* = 45). Statistical significances between the effects of 20 µM (violet) and 50 µM (pink) petasin are indicated black asterisks (****p* < 0.001). Differences between the two heterogeneous membrane models were not statistically significant (indicated by connecting lines and ns in violet and pink colour) at either petasin concentration.

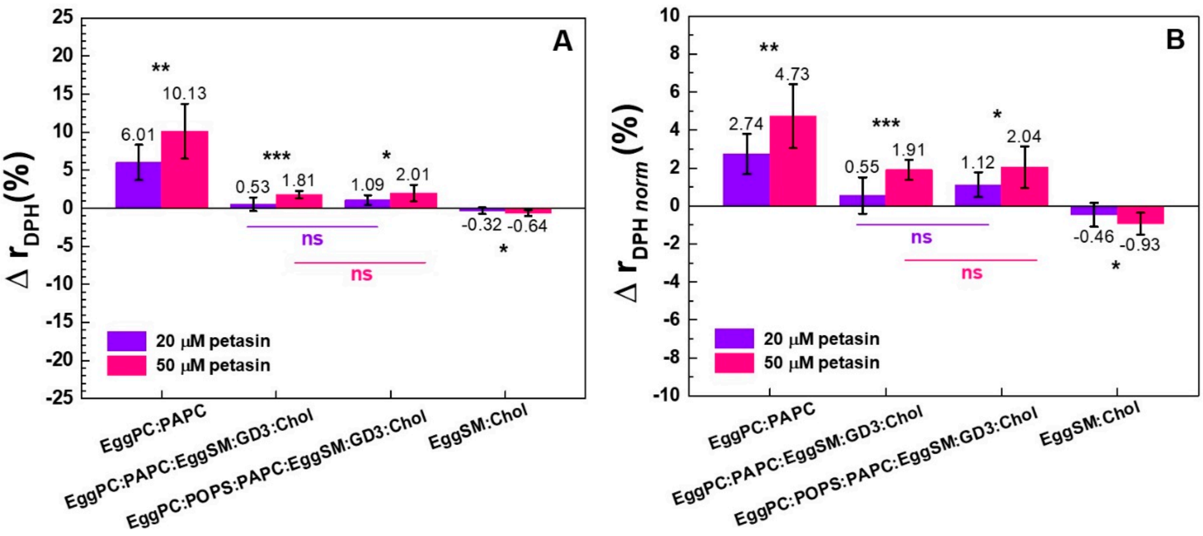

Figure S2. (**A**) Relative (Δr_{DPH}, %) and (**B**) normalized ($\Delta r_{DPH\ norm}$, %) changes in DPH steady-state fluorescence anisotropy induced by petasin in LUVs at 37°C. Relative changes (Δr_{DPH}, %) were calculated with respect to the corresponding DMSO controls, whereas normalized values ($\Delta r_{DPH\ norm}$, %) were scaled to the experimentally defined L_d–L_o phase range (0–100%). Results are presented as mean ± SD (*n* = 18). Statistical significance between the effects of 20 µM (violet) and 50 µM (pink) petasin is indicated by black asterisks (**p* < 0.05, ***p* < 0.01, ****p* < 0.001). Differences between the two heterogeneous membrane models were not statistically significant (indicated by connecting lines and ns in violet and pink colour) at either petasin concentration.

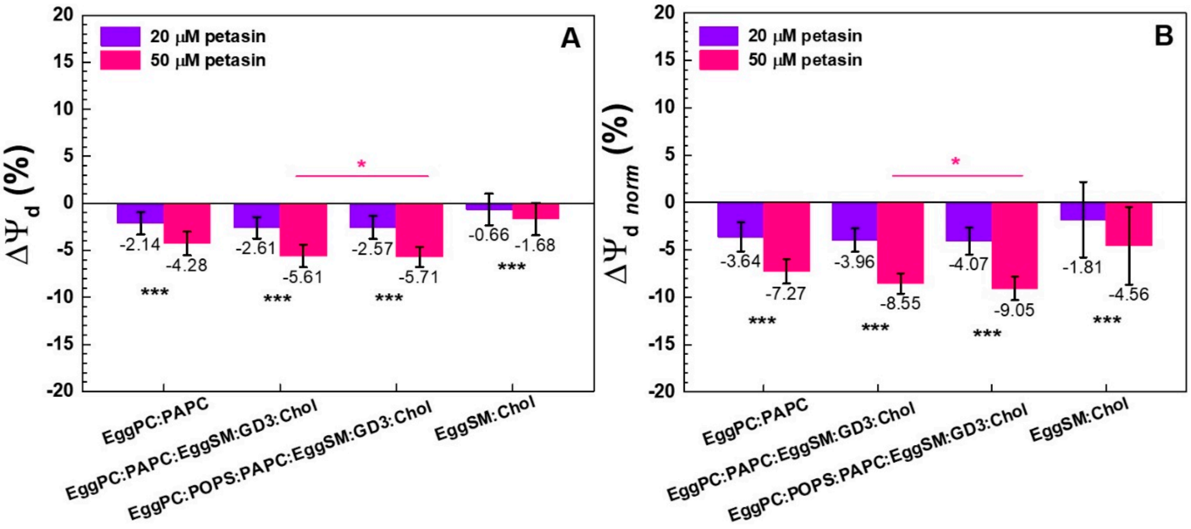

Figure S3. (**A**) Relative ($\Delta\Psi_d$, %) and (**B**) normalized ($\Delta\Psi_{d\ norm}$, %) changes in membrane dipole potential induced by petasin in di-8-ANEPPS-labeled LUVs at 37°C. Relative changes ($\Delta\Psi_d$, %) were calculated with respect to the corresponding DMSO controls, whereas normalized values ($\Delta\Psi_{d\ norm}$, %) were scaled to the experimentally defined L_d–L_o phase range (0–100%). Results are presented as mean ± SD ($n = 45$). Statistical significance between the effects of 20 μM (violet) and 50 μM (pink) petasin is indicated by black asterisks (***$p < 0.001$). A statistically significant difference between the two heterogeneous membrane models was observed only at 50 μM petasin (*$p < 0.05$; indicated by connecting line in pink colour), whereas no significant difference was detected at 20 μM petasin.

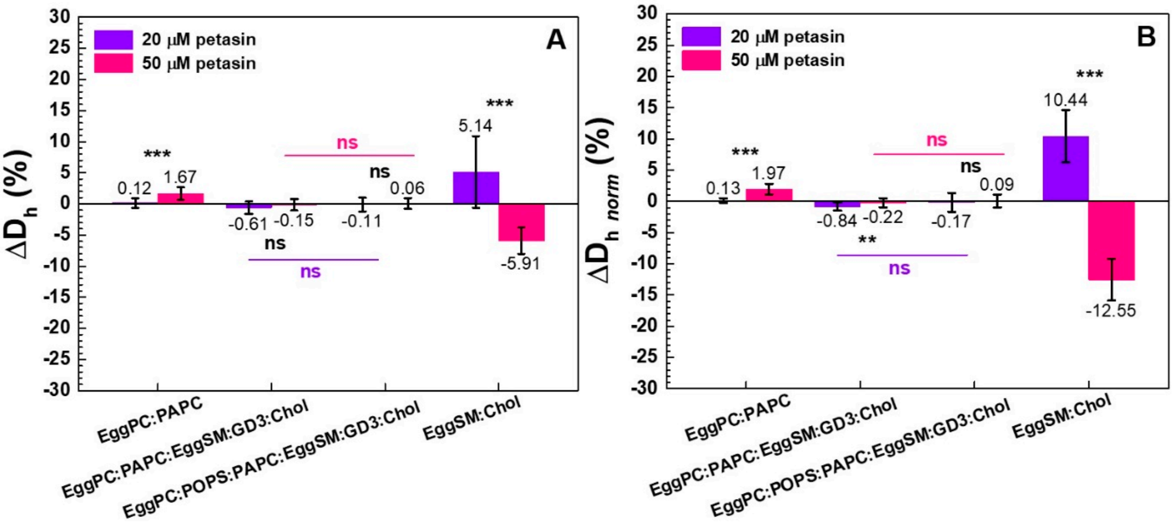

Figure S4. (**A**) Relative (ΔD_h, %) and (**B**) normalized ($\Delta D_{h\ norm}$, %) changes in hydrodynamic diameter induced by petasin in LUVs at 37°C. Relative changes (ΔD_h, %) were calculated with respect to the corresponding DMSO controls, whereas normalized values ($\Delta D_{h\ norm}$, %) were scaled to the experimentally defined L_d–L_o phase range (0–100%). Results are presented as mean ± SD ($n = 18$). Statistical significance between the effects of 20 μM (violet) and 50 μM (pink) petasin is indicated in black colour (**$p < 0.01$, ***$p < 0.001$, ns, not significant). Differences between the two heterogeneous membrane models were not statistically significant (indicated by connecting lines and ns in violet and pink colour) at either petasin concentration.

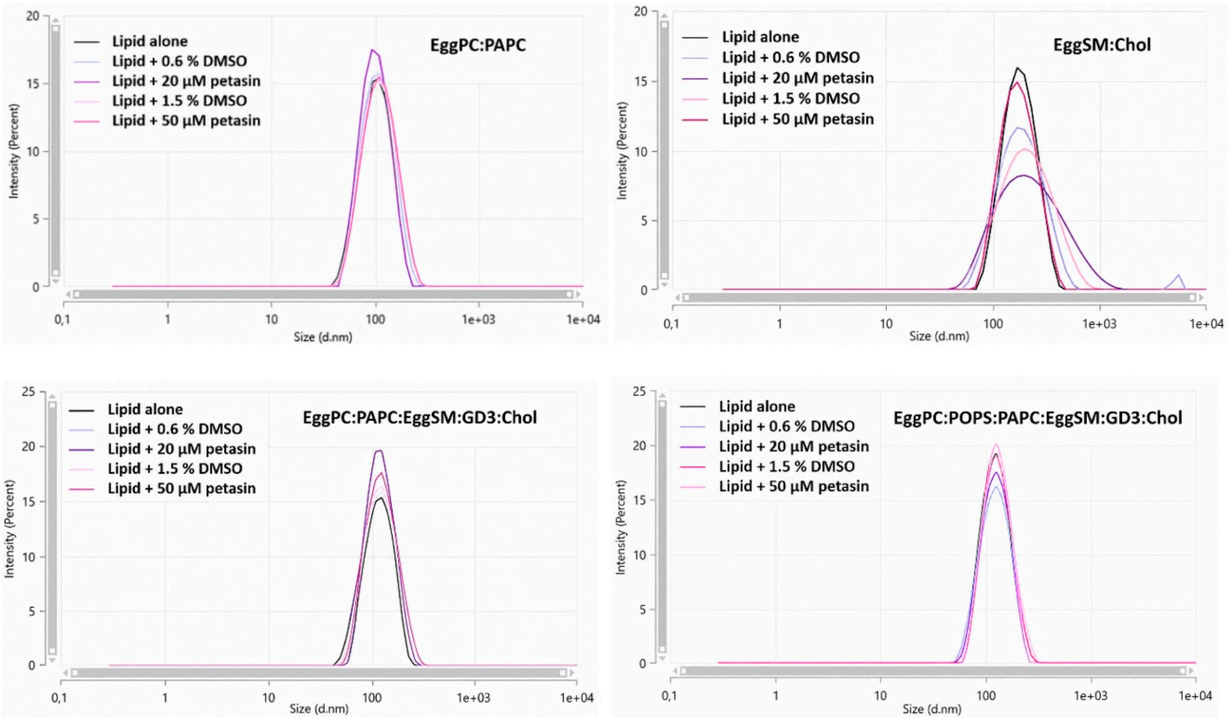

Figure S5. Representative intensity-weighted size distribution profiles of LUVs at 37°C following treatment with DMSO or petasin. Size distributions are presented as intensity-weighted hydrodynamic diameter (D_h) profiles obtained by dynamic light scattering. Reference samples (lipid alone) are shown in black, DMSO controls in light violet (0.6% DMSO) and light pink (1.5% DMSO), and petasin-treated samples in dark violet (20 μM) and dark pink (50 μM). The corresponding quantitative values of D_h and polydispersity index (PDI) are presented in Figure 9A and Table 4.

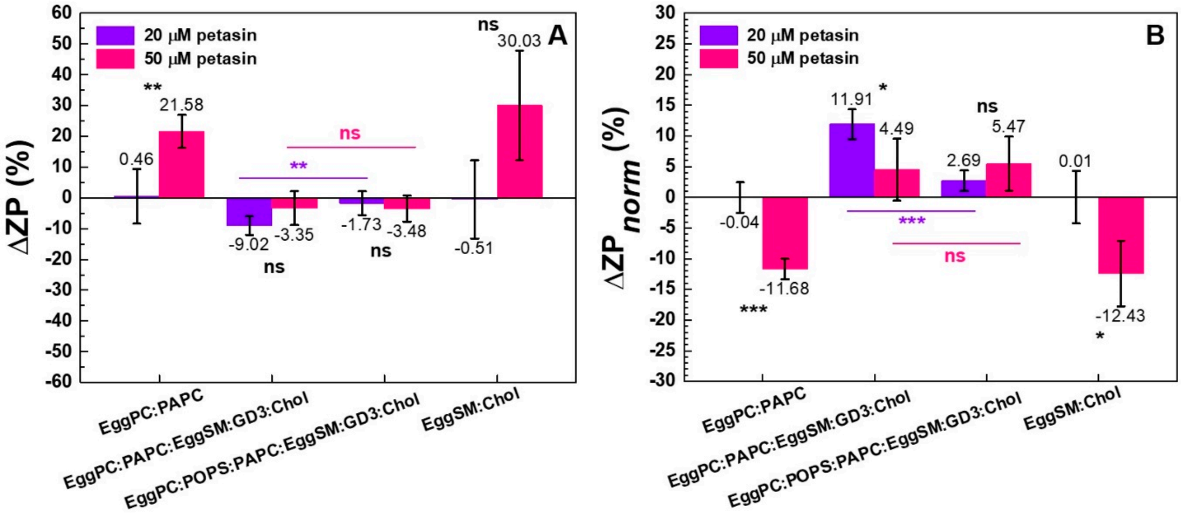

Figure S6. Effect of petasin on the surface electrostatic properties of LUVs at 37°C. Data are presented as (**A**) relative percentage changes in ζ-potential (ΔZP, %) with respect to the corresponding DMSO controls and (**B**) normalized percentage changes (ΔZP_{norm}, %) scaled to the experimentally defined electrostatic window between the least negative EggPC:PAPC (75:25 mol/mol) and the most negative EggPC:POPS:PAPC:EggSM:GD3:Chol (25:5:10:30:10:20 mol/mol) reference ζ-potential values. Thus, normalized values reflect the relative position within the experimentally accessible electrostatic range. Results are presented as mean ± SD ($n = 6$). Statistical significance between the effects of 20 μM (violet) and 50 μM (pink) petasin is indicated in black colour (*$p < 0.05$, **$p < 0.01$, ***$p < 0.001$, ns, not significant). A statistically significant difference between the two heterogeneous membrane models was observed at 20 μM petasin (violet lines and asterisks), whereas no significant difference was detected at 50 μM petasin (pink lines and asterisks).
